# Maintaining physical activity after pain management programmes for people with persistent musculoskeletal pain: A qualitative exploration of barriers, facilitators and activity patterns

**DOI:** 10.1016/j.jpain.2026.106235

**Published:** 2026-05

**Authors:** Gregory Booth, Danielle D’Lima, Lindsay Bearne, Michael Ussher

**Affiliations:** a Therapies Department, Royal National Orthopaedic Hospital NHS Trust, Stanmore, UK; b Department of Population Health & Policy, School of Health and Medical Sciences, City St. George’s, University of London, London, UK; c Centre for Behaviour Change, Department of Clinical, Educational and Health Psychology, University College London, London, UK; d Institute for Social Marketing and Health, University of Stirling, Stirling, UK

**Keywords:** Physical activity, Persistent pain, Musculoskeletal, Pain management programme, Rehabilitation, Barriers and facilitators, Chronic pain, Chronic musculoskeletal pain, Persistent musculoskeletal pain

## Abstract

Many patients with persistent musculoskeletal pain have difficulty maintaining physical activity (PA) long-term following pain management programmes (PMPs). We conducted a qualitative study to explore the barriers and facilitators to maintaining PA long-term after PMPs. We also explored PA patterns that describe trajectories of activity since PMP completion. One researcher conducted semi-structured interviews with 24 people with persistent musculoskeletal pain that completed PMPs, seven partners/spouses of these patient participants, and eight healthcare professionals working on PMPs. The healthcare professionals included four physiotherapists, two occupational therapists and two psychologists. Data were analysed using reflexive thematic analysis and the findings were mapped to the Theoretical Domains Framework. Five themes were generated: (1) Internal drivers for PA maintenance, (2) Fitting PA into life, (3) Symptoms and symptom management, (4) Social networks and influences and (5) Environmental influences. The findings were mapped onto 13 of the 14 Theoretical Domains Framework domains. Four PA patterns were constructed from participants experiences of PA maintenance: (1) Consistently active, (2) Initially consistently active post-PMP but then inconsistently active, (3) Inconsistently active since PMP and (4) Same or a reduction in PA level since the PMP. The findings can inform the development of an intervention to support PA maintenance following PMPs; the intervention can address barriers and facilitators and be tailored to different PA patterns.

## Highlights

• Barriers and facilitators to maintaining physical activity are multifactorial.
• Four unique patterns of physical activity maintenance were constructed.
• We used novel methods to generate interactions between barriers and facilitators.
• Barriers and facilitators map to 13 of 14 Theoretical Domains Framework domains.

**Perspective**

The findings of this qualitative study will be used to develop a physical activity maintenance intervention for people with persistent musculoskeletal pain following pain management programmes.

Alt-text: Unlabelled box

## Introduction

Globally, persistent musculoskeletal pain is the leading cause of disability and the need for rehabilitation,^1^, ^2^ placing a substantial burden on individuals, healthcare systems and society^3^. It is characterised by pain lasting three months or longer in bones, joints, muscles, tendons or other soft tissues.^4^

Many people with persistent musculoskeletal pain who have high disability and pain-related distress attend pain management programmes (PMPs). PMPs are interdisciplinary, group-based interventions (sometimes including 1:1 sessions) that provide rehabilitation and pain self-management support by facilitating behaviour change. A core element of PMPs is supporting the uptake and maintenance of physical activity (PA) due to its many benefits.^5^, ^6^, ^7^, ^8^, ^9^, ^10^ PA refers to any movement produced by skeletal muscles that requires energy expenditure, for leisure, transport, work or exercise.^11^ PA maintenance has been defined as the continued achievement of a personal weekly PA target.^12^

Support following PMPs is often limited to one or two follow-up sessions over 12 months. However, many patients have difficulty maintaining PA levels long-term.^13^ Furthermore, PA interventions for people with persistent musculoskeletal pain, as distinct from PMPs, have not been successful for PA maintenance.^14^ Clearly, maintaining PA is challenging for this population, even after undergoing intensive behaviour change training. Long-term maintenance of PA is essential to sustain the benefits. Therefore, it is important to develop theoretically informed interventions to address this issue.

Uptake and maintenance mechanisms likely differ which could explain why uptake interventions often do not lead to successful maintenance.^15^ PA maintenance interventions, defined as secondary interventions that follow uptake interventions and focus on maintenance, show promise at facilitating PA maintenance.^16^ However, none have been developed or tested for people with persistent musculoskeletal pain who have attended PMPs. A specific PA maintenance intervention for this population is needed due to their likely unique barriers and facilitators to maintaining PA following PMPs. However, to our knowledge, no previous studies have explored these barrier and facilitators. This is essential to develop effective theoretically informed behavioural interventions.^17^

The Theoretical Domains Framework (TDF) defines 14 domains which comprehensively represent modifiable determinants of behaviour and can be used to categorise barriers and facilitators to behaviour.^18^ It is particularly useful for complex multifactorial health behaviours such as PA maintenance. We conducted a systematic review using the TDF to identify prominent barriers and facilitators to PA in people with persistent musculoskeletal pain. We could not identify which TDF domains, barriers or facilitators are more prominent for PA uptake versus maintenance, as these different phases were not explicitly explored in any of the studies. Furthermore, none of the studies included people who had completed PMPs.^19^

There are many potential barriers and facilitators that could be targeted in PA maintenance interventions and it is important to identify which are the most influential.^17^ To support this, we identified interactions between barriers and facilitators (i.e., how barriers and facilitators can influence each other), considering it efficient to focus on barriers and facilitators with a broad influence.

Studies have also identified different patterns of maintenance that may have consequences for intervention development.^12^, ^20^ For example, three PA patterns were identified in people living with and beyond gastrointestinal cancer: maintained PA, intermittent PA and low PA.^20^ It is possible that interventions can be made more effective by tailoring interventions to a person’s pattern.

The PAMPER study is a mixed-methods study that aimed to develop an intervention to support PA maintenance after PMPs for people with persistent musculoskeletal pain.^21^ This study aimed to explore the barriers, facilitators and patterns of PA maintenance in people with persistent musculoskeletal pain, following PMPs, to inform the development of a theoretically grounded, contextually tailored intervention to support PA maintenance.

## Methods

### Study design

This qualitative study is within a pragmatist paradigm, as it generated findings that will be used to develop an intervention aimed at improving healthcare and outcomes from clinical practice. Ethical approval was obtained from the North West–Liverpool Central Research Ethics Committee and the UK Health Research Authority on 4th June 2024 (REC reference: 24/NW/0174, IRAS Project ID: 340674). The protocol has been published^21^ and registered (https://osf.io/jq95b). This study is reported consistent with the Consolidated Criteria for Reporting Qualitative Research (COREQ) checklist.^22^

### Participants

#### Inclusion criteria

• People with persistent musculoskeletal pain who were between six- and 18-months post completion of one of the participating PMPs at the time of study invitation. This duration enabled participants to have experience of attempting PA maintenance and has been used in previous qualitative studies on PA maintenance.^20^, ^23^
• PA partners of patient participants (i.e., people that support them with PA (e.g., encourage them) or that they do PA with, either formally or informally).
• Healthcare professionals (HCPs) who work on one of the participating PMPs and facilitate PA during the programme (commonly physiotherapists, occupational therapists and psychologists).

### Exclusion criteria

• People with persistent musculoskeletal pain who self-report having a condition where moderate-vigorous PA is contraindicated (e.g., uncontrolled cardiac conditions).
• People with persistent musculoskeletal pain that are pregnant (pregnancy affects PA levels and would require a separate intervention).

A purposive sampling strategy was used. This ensured equal representation of individuals who were either maintaining or not maintaining a consistently higher amount of weekly PA each before their PMP. PA maintenance status was self-reported during screening. We also aimed for maximum variation in other participant characteristics (including diagnoses, duration of pain, age, gender, ethnicity, educational qualifications, employment status, PMP completed, time since PMP and whether they have a PA partner). The sample variation was monitored by iterative review after each participant was recruited. Regular research team meetings during recruitment helped to focus our efforts towards recruiting participants with more varied characteristics. No sampling criteria was used for PA partners as they were nominated to participate by participants with persistent musculoskeletal pain. We also prioritised representation of different HCPs and different PMPs.

Sample size was guided by the principles of information power.^24^ The studies aim and use of the TDF to inform the interview topic guide increased the information power. The closeness of the participant groups with PA maintenance following PMPs increased information power. However, the inclusion of multiple groups and maximum variation sampling increased our sample size requirements. The cross-case analysis strategy and generation of PA patterns also increased sample size requirements. After considering these principles, we anticipated requiring 24 patients: 12 maintaining consistently higher PA than before their PMP and 12 not maintaining. We aimed for eight to 12 HCPs. These principles were regularly reviewed throughout recruitment and data generation to determine the final number of participants required to achieve sufficient information power. Recruitment stopped when the research team agreed sufficient power had been achieved.

### Setting and recruitment

Participants were recruited from three National Health Service PMPs in the UK, selected to enhance geographical variation. One North London PMP sees patients from across the UK. Another PMP, in Central London, sees patients primarily in central and south London and southeast England. Finally, a Liverpool-based PMP sees patients from across northwest England.

Patients and HCPs were invited to take part by email, including a Participant Information Sheet, by a member of the site’s clinical team. If they were interested, they emailed the researcher (GB). GB called interested patients to discuss the study, answer questions and screen them. During screening, patients were asked about their musculoskeletal pain diagnoses, pain duration, location of pain, which PMP they attended, time since PMP completion, consistency with PA maintenance since PMP, age, gender, ethnicity, educational qualifications, employment status and whether they have a PA partner. Interested HCPs were emailed a screening questionnaire asking about their profession, PMP working on, time working on PMPs, grade and gender. They were asked to return the questionnaire by email. GB offered interested HCPs the opportunity to discuss the study by email or phone if they had questions.

If the patients and HCPs were eligible, interested, and selected for interview (aligning with maximum variation sampling), they were sent a consent form (by email or post, as preferred), which they returned.

If a patient participant wanted a PA partner to participate, they were sent a PA partner Participant Information Sheet and consent form to give to their partners. Partners were advised to contact the researcher if they had any questions and completed the consent form.

### Data generation

Semi-structured interviews were conducted by GB between July 2024 and January 2025. HCPs and patients without PA partners participated in individual interviews. Patients with PA partners had dyadic interviews. Patient participants were asked about their experiences, and PA partners and HCPs were asked for their perspectives on what influences the patient. GB took field notes during the interviews, kept a reflective diary, and produced a summary following each interview, identifying potential changes to the topic guide.

Interview topic guides were drafted by the researchers and reviewed by the PAMPER patient and public involvement (PPI) group and revised accordingly. The patient/PA partner topic guide was piloted with two PPI representatives and amended. Further feedback on the HCP topic guide was provided by a member of the PAMPER Steering Group. Both topic guides were iteratively refined throughout data generation, following discussions within the study team.

Interviews began with open questions regarding participants’ experiences and their perceived barriers and facilitators of PA maintenance. The following questions were informed by the 14 TDF domains to ensure comprehensive exploration of potential barriers and facilitators.^18^ Domains were grouped when questions relating to them were similar to avoid repetition and enhance natural flow, rather than asking about each domain independently (e.g., Knowledge and Beliefs about Consequences). The TDF related questions were only used if a participant had not raised barriers and/or facilitators relating to a domain in the initial open section of the interview or if follow-up relating to a domain was required. Beginning the interviews with open questions enabled participants to explore topics unrelated to the TDF domains and to facilitate understanding of relationships between barriers and facilitators.^25^ Topics are summarised in Table 1 and an iteration of the topic guides is displayed in supplementary material.

**Table 1:** Interview topic guide topics.

**Topic guide topics**	
**Patient and PA partner topic guide**	**Healthcare professional topic guide**
Current PA (activities, location and level)	Perceptions of how well patients maintain PA after a PMP
Experiences of maintaining PA since PMP	Patients’ feelings about maintaining PA long-term
Feelings about maintaining PA long-term	Barriers and facilitators to patients maintaining PA long-term, after a PMP
Barriers and facilitators to maintaining PA since PMP	
Sections relating to TDF domains: − Planning, intentions and goals towards PA *(TDF domains: Intentions, Goals, Reinforcement and Behavioural Regulation)* − Confidence related to long-term PA *(TDF domains: Skills, Beliefs about Capabilities and Optimism)* − Feelings when doing PA *(TDF domain: Emotions)* − Problem solving, memory regarding PA and PA monitoring *(TDF domains: Skills; Memory, Attention & Decision Processes; Behavioural Regulation)* − Knowledge and beliefs about maintaining PA *(TDF domains: Knowledge and Beliefs about Consequences)* − Social influences and *communication (TDF domains: Skills and Social Influences)*	Sections relating to TDF domains: − Planning, intentions, routine, competing demands, burden of PA *(TDF domains: Intentions; Goals; Optimism Reinforcement, Behavioural Regulation, Memory, Attention & Decision Processes; Environmental Context and Resources; Social/Professional Role and Identity)* − Confidence related to long-term PA *(TDF domains: Beliefs about Capabilities and Optimism)* − Problem solving, memory regarding PA and PA monitoring *(TDF domains: Skills, Behavioural Regulation and Memory, Attention & Decision Processes)* − Knowledge and Beliefs about maintaining PA *(TDF domains: Knowledge and Beliefs about Consequences)* − Social influences and communication about PA *(TDF domains: Social Influences and Skills)* − Other support – *healthcare* professionals and community-based PA *(TDF domain: Environmental Context and Resources)*

Abbreviations: PA: physical activity; TDF: Theoretical Domains Framework

Interviews were conducted either in-person on PMP premises, by video call using Microsoft Teams, or by telephone, depending on participant preference. Participants were encouraged to move around, change positions or take breaks if required. They were also advised they could stop the interview at any time. No researchers were known to the participants prior to the study, aside from GB, where several of the participating HCPs were his colleagues. The participants were told the lead researcher’s background during initial discussions about taking part and reminded prior to the interview. Steps were taken to mitigate potential power imbalances between GB and the HCPs he had existing professional relationships with. These included explicit role clarification, adopting a non-judgemental interviewing style, assuring confidentiality and ongoing reflexive practice.

The interviews were audio recorded using a digital audio recorder and transcribed verbatim using Otter.ai artificial intelligence and machine learning software (Otter.ai, Inc). Confidentially was ensured by uploading audio files to Otter named with study identification numbers only. Audio files and transcripts were deleted from Otter once transcripts had been downloaded. To ensure accuracy, recordings were listened to and transcripts edited as required. No ambiguities were identified that needed any transcripts to be checked with interviewees.

In addition to details of participant’s demographic and clinical characteristics collected during screening, participants completed a questionnaire before the interview, to describe the sample, including marital status; who they live with; comorbidities for people with persistent musculoskeletal pain; and age, gender, ethnicity, employment, educational qualifications and health conditions for PA partners.

### Data analysis

The pragmatism paradigm permits methodological flexibility and integration of different analytical methods and tools. It enabled the combination of reflexive thematic analysis, used to generate rich contextual understanding of PA maintenance, with the TDF, a practically useful tool that is part of the Behaviour Change Wheel intervention development framework that is guiding the PAMPER study.^17^, ^26^ Analysis was conducted in three phases:(i) a reflexive thematic analysis to generate themes and subthemes;^27^ (ii) subthemes were mapped to the TDF;^18^ (iii) interactions between different subthemes and, subsequently, TDF domains, were mapped out.

Step 1: reflexive thematic analysis

Qualitative data relating to barriers and facilitators of PA maintenance were initially analysed inductively, using reflexive thematic analysis to generate themes and subthemes.^27^, ^28^ Subthemes were generated to maintain the detail relating to each barrier and facilitator and enable TDF mapping at a more granular level to support intervention development. The theoretical flexibility offered by reflexive thematic analysis allowed for a critical realist ontological perspective, acknowledging that reality exists independently of our perceptions but is mediated through language and context.^28^ It also allowed a contextualist epistemology that enabled generation of an understanding of barriers and facilitators that is contextualised to each participant. Aligning with reflexive thematic analysis, a contextualist epistemology acknowledges that the researcher’s values and practices shape the knowledge they generate. We also assumed that the dyadic partnerships and participants’ knowledge of the researcher’s background and aim of the study would impact the data generated and subsequent analysis. Therefore, it was important to acknowledge and reflect on the potential impact of these influences and subjectivity, while harnessing it for data generation and analysis.

Regarding reflexivity, the lead researcher (GB) is a specialist pain physiotherapist and physically active white male, who conducted this study for their PhD. As a pain physiotherapist, GB was an insider with HCPs and an outsider to patients and PA partners. As an insider, GB’s clinical experience aided understanding but risked influencing interpretation of the data. GB’s outsider perspective helped to notice the differences in patient’s barriers and facilitators but could have led to misinterpretation of the data. Team meetings helped consider alternative interpretations of the data, aided by two other researchers (DD and MU) reading three of the early transcripts and contributing to the analysis at multiple other stages. LB has a background in physiotherapy and rehabilitation and both MU and DD have backgrounds in behavioural science.

GB familiarised himself with the data by concurrently listening to audio recordings and reading and correcting transcripts. Anonymised transcripts were then uploaded to QSR NVivo 14 software.^29^ GB then coded (assigning descriptive labels to data fragments) all transcripts in NVivo using a bottom-up, data-driven (inductive) approach, with the aim of staying close to participants’ own words and meanings. Codes were also created to highlight interactions between barriers and facilitators (e.g., when a participant described a barrier or facilitator influencing a different barrier or facilitator). Codes for all participant groups were kept together, as we aimed to generate knowledge of barriers and facilitators from all perspectives, but not to compare them, as the focus was on the patients’ PA. During coding, the dyadic interviews were treated as single units of analysis as the PA partners typically elaborated on, confirmed or challenged the patient’s perspective, thereby co-constructing a shared narrative.

Codes representing similar barriers and facilitators were clustered together and initial themes and subthemes were constructed. Themes and subthemes were then further developed and refined iteratively by critically reviewing them and considering alternatives. During coding and theme generation the researchers had multiple meetings to consider alternative data interpretations and theme/subtheme formation. Data/codes relating to patient participants’ experiences of maintaining PA were used to construct PA patterns by grouping participants together that described similar experiences and trajectories of PA. These patterns were discussed critically by the researchers and refined into a set of distinct patterns.

Step two: mapping subthemes to the TDF

Two researchers (GB and DD) independently mapped the subthemes generated in step one to the TDF^18^ and met to reach consensus for the mapping. MU reviewed and agreed with the mapping. We then flipped the analysis to view the subthemes within each TDF domain. This enabled us to see which TDF domains are most prominent. This could affect future intervention design by informing which TDF domains may be more important to focus on.

Step three: mapping interactions between different subthemes and then TDF domains

Codes regarding interactions between barriers and facilitators (created in step one) were mapped onto the subthemes to generate interactions between subthemes. This enabled us to generate understanding of how subthemes (representing groups of barriers/facilitators) can influence others. The direction of influence (positive or negative) was then identified from the quotes corresponding to each code. We referred to the subtheme to TDF mapping, conducted in step two, to see how TDF domains can influence each other. We then explored the number of data fragments within each code and the number of participant quotes expressing significance of each interaction to consider which may be more significant. We aligned our approach to considering what constitutes a significant interaction with that of theme generation, with each interaction needing to be expressed by enough participants to be considered a pattern and including statements of significance in the corresponding data. This resulted in a minimum of four participants expressing each significant interaction in our study.

### Exploring subtheme relationship with PA patterns

Following the generation of PA patterns in step one and the mapping of subthemes to the TDF in step two, we identified how many participants within each pattern had data coded to each subtheme. The data was then reviewed, and we recorded how many participants within each pattern described a barrier, facilitator or both within each subtheme.

### Patient and public involvement

PPI was integral to the design and conduct of this study. In addition to their influence on topic guide development and pilot interviews (described above), they identified the need to include PA partners in dyadic interviews, reviewed all participant-facing materials and helped write the lay results summary disseminated to study participants.

## Results

Across the three sites 271 potential participants were invited to participate: 208 were patients and 63 were HCPs. Twenty-eight patients and nine HCPs expressed interest and were screened and eligible. Twenty-six patients and all nine HCPs were offered an interview. Twenty-four patients (seven with partners) and eight HCPs consented and were interviewed, making a total of 32 interviews. Twenty-five interviews were video calls (19 patients, six HCPs), four were telephone calls (all patients) and three were in-person (one patient, two HCPs). Patient interviews averaged 60 min (range 39–89 min) and HCP interviews averaged 47 min (36–60 min).

After 32 interviews, the research team deemed we had achieved sufficient information power as the purposive sampling criteria were achieved and the interviews provided in-depth data regarding many different barriers and facilitators to PA maintenance, and were generating minimal new conceptual insights.^24^ The final number of 24 patients and eight HCPs correspond with our initial estimates of the sample required to achieve sufficient information power.

Characteristics of patient participants are displayed in Table 2, PA partners in Table 3 and HCPs in Table 4.

**Table 2:** Participant characteristics – people with persistent musculoskeletal pain

**n**	24
**Gender (n, %)**	
Female	16 (67%)
Male	7 (29%)
Non-binary	1 (4%)
**Age (years) median (IQR)**	54 (34–65.5)
**Ethnicity (n, %)**	
White	21 (88%)
Asian	1 (4%)
Black British	1 (4%)
Latin American	1 (4%)
**Highest educational qualification (n, %)**	
GCSE (or equivalent)	3 (12.5%)
A-level (or equivalent)	9 (37.5%)
Degree (or equivalent)	12 (50%)
**Employment (n, %)**	
Working (paid employment)	10 (42%)
Studying	4 (17%)
Volunteering	1 (4%)
Not working due to pain	7 (29%)
Not working due to other reason	2 (8%)
Retired	2 (8%)
**Living circumstances (n, %)**	
Living alone	3 (12.5%)
Living with spouse or partner	15 (62.5%)
Living with others	6 (25%)
**Physical activity status (n, %)**	
Consistent	12 (50%)
Inconsistent/inactive	12 (50%)
**Physical activity partner (n, %)**	
Yes	13 (54%)
No	11 (46%)
**Multiple diagnoses (n, %)**	13 (54%)
**Musculoskeletal pain conditions (n, %)**	
Fibromyalgia	10 (42%)
hEDS/HSD	5 (21%)
Persistent low back pain (with and without leg pain)	10 (42%)
Persistent thoracic spine pain	1 (4%)
Persistent neck pain (with and without arm/hand pain)	3 (12.5%)
Scoliosis	3 (12.5%)
Persistent peripheral joint pain	4 (17%)
Osteoarthritis	8 (33%)
Rheumatoid arthritis	2 (8%)
Ankylosing spondylitis	1 (4%)
**Previous orthopaedic surgery**	5 (21%)
**Number of pain sites (n, %)**	
1	0
2	1 (4%)
3	2 (8%)
4	1 (4%)
5+	20 (84%)
**Pain duration (years) median (IQR)**	11 (5–24.5)
**Comorbidities (n, %)**	
Yes	20 (83%)
No	4 (17%)
**Comorbidity type (n, %)**	
Mental health	6 (25%)
Gastrointestinal	6 (25%)
Cardiovascular	7 (29%)
Raynauld’s disease	2 (8%)
Autonomic	4 (17%)
Neurodiversity (ASD/ADHD)	3 (12.5%)
Gynaecological	3 (12.5%)
Migraine/headache	4 (17%)
Thyroid	3 (12.5%)
Eye	3 (12.5%)
Other	7 (29%)
**Time since PMP completion (months) mean (range)**	11.5 (6−17)

NB: employment figures add up to more than 24 as some participants had 2 status’ (e.g., working (paid employment) and studying. Musculoskeletal/pain conditions and comorbidities add to up more than 24 as multiple participants had multiple conditions.

Abbreviations: ADHD: attention deficit hyperactivity disorder; ASD: autism spectrum disorder; hEDS: hypermobile Ehlers-Danlos Syndrome; HSD: hypermobility spectrum disorder; IQR: interquartile range; PMP: pain management programme

**Table 3:** Participant characteristics – physical activity partners.

**n**	7
**Gender (n, %)**	
Female	2 (29%)
Male	5 (71%)
**Age (years) median (IQR)**	59 (27−72)
**Ethnicity (n, %)**	
White	6 (86%)
Asian	1 (14%)
**Highest educational qualification (n, %)**	
GCSE (or equivalent)	1 (14%)
A-level (or equivalent)	3 (43%)
Degree (or equivalent)	3 (43%)
**Employment (n, %)**	
Working (paid employment)	5 (71%)
Retired	2 (29%)
**Health conditions (n, %)**	
Yes	6 (86%)
No	1 (14%)
**Health condition type (n, %)**	
Musculoskeletal	3 (43%)
Cardiovascular	2 (29%)
Respiratory	2 (29%)
Skin	2 (29%)
Gastrointestinal	2 (29%)
Diabetes	1 (14%)

**Table 4:** Participant characteristics – healthcare professionals.

n	8
**Profession**	
Physiotherapist	4 (50%)
Occupational therapist	2 (25%)
Psychologist	2 (25%)
**Gender (n, %)**	
Female	7 (87.5%)
Male	1 (12.5%)
**Grade (NHS Agenda for Change band) (n, %)**	
7	5 (62.5%)
8a	1 (12.5%)
Split 7/8a	1 (12.5%)
8c	1 (12.5%)
**Duration working on PMPs (years) median (IQR)**	13.5 (4–19.5)

Abbreviations: IQR: interquartile range; NHS: National Health Service; PMP: pain management programme.

### Main analysis

Five themes were generated, with multiple subthemes. Subthemes were mapped onto 13 of the 14 TDF domains. See Table 5 for quotes relating to each subtheme and subtheme to TDF mapping. See Table 6 for subthemes organised by TDF domain. As is common and recommended in reporting the findings of reflexive thematic analysis,^28^ qualitative descriptors such as “all”, “almost all”, “many”, “some” and “a few” are used to indicate the frequency with which barriers and facilitators were referred to by participants. These descriptors are intended to aid interpretation by indicating the prominence of particular barriers and facilitators, rather than implying numerical distribution or representativeness. We also consider that data may be highly meaningful even if few participants mention it.

**Table 5:** barriers and facilitators themes and subthemes with quotes and TDF mapping.

**Theme**	**Subtheme**	**Participant quotes**	**TDF domains**
Internal drivers for physical activity maintenance	Feelings around PA maintenance	*“I think I didn't realize how much fear I had […] And now I notice it, and so I can correct it. So when I sit and my feet turn inwards and I start scrunching them up, I'm aware of it, and I'll I've got [name of physio] the physio, I've got his voice in my head, like your feet flat on the floor, natural position, and I will talk out loud to myself as well. And you know, I'm moving myself to put, sit myself up straight and walk heel toe rather than dragging. So I'm aware that the fear is there, but I am then I'm able to talk it down, discount it.” (Patient 16)*	**Emotion;** **Skills**
	Beliefs about the value of PA maintenance	*“And the other thing that I think has kept me motivated is because I've actually noticed a reduction in my pain as a result of this, like, and that I think has been such a big, it’s when I don't end up going for whatever reason, I get, like, really worried that my pain is going to come back, like it is still there, but that it's going to get worse again, and like that all of this progress, I think I'm still quite worried about that, that I'm going to backslide. And so feeling like I'm actually getting the pain is getting less is been a huge motivating factor” (Patient 6)*	**Beliefs about Consequences;** **Knowledge**
	Believing in ability to maintain PA	***“**Sometimes I feel quite confident. And then other days, like, like, on Friday, I did like, some exercises and over the weekend, like my the backs of my shoulders were like, quite like, irritated. And then it kind of makes me feel not as confident as I have been, and it kind of gets you down a bit. And so that's why I went today. And even though I didn't focus on that group, I was just cautious of it […]I know for a fact that when I'm in pain, or I've got pain, which might be a result of deconditioning, I'm not as confident or, you know, I don't tackle things, or I shy away from things, so it's important that I keep it up. And, yeah, so when I don't, when I'm not able to do it, it can make me feel that, you know, dejected.” (Patient 23)*	**Beliefs about Capabilities;** **Knowledge**
	Goals for PA maintenance	*“I was quite open about this at the pain clinic that I am trying to have a baby, and so that has been a huge sort of motivating factor, because I would be a solo mom, and being able to care for a child with the sort of pain levels that I had before would have just been much more difficult. And so that's really been a huge motivating factor, and also why I'm really pleased to see improvements in both my mobility and pain levels, because it's made me feel sort of stronger.” (Patient 6)*	**Goals;** **Emotion**
	Remembering the past and accepting the now	**Researcher:***“What if someone, if someone's been very active before, say, before they got pain? How do you think that affects them with their sort of long-term physical activity?”***Participant:***“I think that they then, they find it really hard to be satisfied with the level of activity they've done. I think they experience loss essentially […] sometimes I find they're much happier when they do something completely different.” (HCP 5)*	**Emotion;** **Social/Professional Role and Identity;** **Beliefs about Capabilities**
	Keeping track and checking in on PA	*“We're very lucky where we live, say, at the bottom of the road is the beach, and it's there's lots of benches. So, I measure my progress in in benches. How many benches I can get to, either before I need to sit down and rest, or, you know, I've got to that bench today, and I could only get to that one the other day. So that's really helpful for for me, is where, where we actually live.” (Patient 16)*	**Behavioural Regulation;** **Reinforcement**
Fitting physical activity into life	Competing demands and life roles and responsibilities	*“A patient can leave the program with the best intentions, but then life comes along and trips them up. Ill health, family commitments, change of role, work, moving. When life trips us up, often, the first thing to change is focus on the goals or themselves. They'll focus on kids, work, the dog, husband, whatever. And so there needs to be this sort of resetting.” (HCP 8)*	**Environmental Context and Resources;** **Social/Professional Role and Identity;** **Social Influences**
	Habits, routine and memory	*“Well, it is my day-to-day life, so I think it just, is it. I don't really think it's sort of fitting into it. I think it's, well, it is fitting in, but you make it fit. So like, I've made cooking fit because I love cooking, and I've made the walks fit, because I need to pick up my prescription once a month. Going outside always, always makes you feel better, even if it's just, you know, once or twice a week, it will reset you and make you feel better. You know, stretching I have to do, otherwise my hips can lock up or my knees can lock up. So it has to be done. But, you know, yeah, I sort of, I just sort of find it fits in naturally. It's not like you have to force it. If you have to force it, it don't work.” (Patient 8)*	**Reinforcement;** **Behavioural Regulation;** **Memory, Attention and Decision Processes**
	Planning	*“As soon as I get up, I've got a plan, you know, like I start moving around. I walk around my bungalow. I've got an upstairs, and I try and sort of go up and down stairs every day if I can do it. So, yeah, I do sort of try and plan, but then, you know, it depends on the degree of pain, because if you can't sort of walk properly, you know, if your pain is that bad, you're holding on to everything, there's no way you can go upstairs, because it's dangerous. But I do sort of walk around bungalow. I won't just sit about all day.” (Patient 12)*	**Behavioural Regulation;** **Intentions**
	Other health conditions	*“It's everything about the mental health like because if I have pain, I feel grumpy, I feel sad, and I feel anxious to go outside because I could probably hurt myself, and I feel shame, because people are going to see me with the walking stick, you know.” (Patient 10)*	**Beliefs about Capabilities;** **Skills (Physical);** **Emotion**
	Adapting PA, flexibility and resilience	*“You know, if you can't get out of the house, you can still do some exercises at home… But I would argue that part of that probably comes down to their mindset again, and whether actually patients feel confident enough to think outside the box” (HCP 3)*	**Skills;** **Behavioural Regulation**
Symptoms and symptom management	Influence of persistent musculoskeletal pain symptoms	*“The thing that affects the physical physical activity is that there's finding activities that aren't very quickly painful. So say just, just walking to the bus stop. It's, it's a question of 50–100 yards, but then having to stop, and by the time I get to the bus stop, well [PA partner] would have seen it this afternoon, coming here, going to the first bus, I was really hobbling, and you could see that.” (Patient 14)*	**Beliefs about Capabilities;** **Skills (Physical)**
	Symptom management	*“I had to build it up. I mean, when I came off the program, I was still getting pain in my legs, but not as much, I knew how to deal with it. You know, if it's like, you've got to pace yourself, you know that that was a big thing from the pain management course was about pacing. Yeah, you know, don't, don't overdo it. Don't bust, you know. Just know, and knowing that if you did sort of overdo it, you just start again. Go back to, you know, stage one kind of thing, and then build it up. And build it up.” (Patient 15)*	**Skills;** **Behavioural Regulation**
	Adapting PA in response to symptom level	*“For me, it's been psychological. It's the fact that if I can't today I’ll try again tomorrow. It is the pacing for me has been a game changer. So I will consistently do something, even if it is only two 10 min walks around the corner. You know even if it’s going up down the stairs that extra time I’ll do something, whether it's small or you can do it in the armchair.” (Patient 13)*	**Skills;** **Behavioural Regulation**
Social networks and influences	Encouragement, motivation and positive emotional responses	**Patient: “***Mum's always been very much, even from the start when I was really poorly and couldn’t get out there, she she was always trying to encourage me to go up for walks or go on a treadmill, for example, and stuff like that. And she likes the fact that we do Pilates as well, doesn't she? Because I think she's also seen a change in me”***Partner:** “*She likes that you're doing it as well.”***Patient:** “*Yeah, she likes that I'm actually getting out of the house and doing that sort of thing.” (Patient 4 and PA partner)*	**Social Influences**
	Practical support	*“I am still trying very hard not to go out unless I know I'm meeting him somewhere, so that I fall over, sometimes even with them, but then they're there to steady me. If I'm holding [PA partner] hand, say he will steady me.” (Patient 17)*	**Social Influences**
	Negative influence of others	*“A lot of patients have got people in there who have got pain and their beliefs are quite fixed. And you know, when someone's beliefs are fixed, you're not, you're not going to budge them. And you know, just have to accept that. But if they've got somebody there saying, oh, everyone in the family has had a bad back, you should avoid this. You know, it’s going to be more challenging for that person to carry on with exercise, for sure, isn't it, you know?” (HCP 7)*	**Social Influences**
Environmental influences	Location of PA	**Patient*****:** “I've just got to keep going to see the birds in botanic and going to the park with them, you know, with these 2 [partner and dog]. Yeah, I mean, we moved here and we've not even been on the beach, yeah, we might do that next year. Yeah, we'll look forward to that. A big goal, yeah, a big goal. Yeah, yeah, there's some nature reserves nearby as well. So that's good.”***Partner: “***Well we back on, our house virtually, virtually backs onto an RSPB. It's the marshes, basically. So if you're into your birds and which were we are.”***Patient:** “*Yeah, spotting all sorts, 1000s of geese.”***Partner: “***Yeah, it's just, it's just a way of, it really, I think, helps both our mental health, yeah, for sure.” (Patient 15 and PA partner)*	**Environmental Context and Resources**
	Financial influences	*“I think people can, I have heard people access different services, especially when they have the money to do so. But there's, because public pools or gyms require cost […] So I think there's a financial part here where we, some people are not accessing the same for financial reasons.” (HCP 1)*	**Environmental Context and Resources;** **Intentions**
	Organised PA opportunities	*“It's where to go, like I would like to go a gym, but not a gym as I know it, sort of thing. It would be good to have classes with people in a similar situation to me, like I did on the pain management. Everyone had some type of pain but was all different. It was better paced I felt. Whereas as now you just go in the gym, it's soulless, isn't it? It's all the machines, and some of them I just can't do, my grips not great and stuff like that. And I just think it's a bit intimidating, to be honest with you, yeah, and even some of the yoga classes, whether you go on, you know, the beginners or medium, whatever, that can be a bit much.” (Patient 13)*	**Environmental Context and Resources;** **Social Influences**
	Weather	*“Yeah, the pain definitely makes my like, the cold makes the pain in my joints worse. If it's too cold, then like, my joints are already telling me, yeah, we're not doing that today.” (Patient 18)*	**Environmental Context and Resources**

**Key:** HCP: healthcare professional; PA: physical activity.

**Table 6:** Subthemes organised by TDF domains.

**TDF domain**	**Subthemes mapped to domain**
**Knowledge**	• Beliefs about the value of PA maintenance • Believing in ability to maintain PA
**Skills**	• Feelings around PA maintenance • Other health conditions • Adapting PA, flexibility and resilience • Influence of persistent musculoskeletal pain symptoms • Symptom management • Adapting PA in response to symptom level
**Social/Professional Role and Identity**	• Remembering the past and accepting the now • Competing demands and life roles and responsibilities
**Beliefs about Capabilities**	• Believing in ability to maintain PA • Remembering the past and accepting the now • Other health conditions • Influence of persistent musculoskeletal pain symptoms
**Beliefs about Consequences**	• Beliefs about the value of PA maintenance
**Reinforcement**	• Keeping track and checking in on PA • Habits, routine and memory
**Intentions**	• Planning • Financial influences
**Goals**	• Goals for PA maintenance
**Memory, Attention and Decision Processes**	• Habits, routine and memory
**Environmental Context and Resources**	• Competing demands and life roles and responsibilities • Location of PA • Financial influences • Organised PA opportunities • Weather
**Social Influences**	• Competing demands and life roles and responsibilities • Encouragement, motivation and positive emotional responses • Practical support • Negative influence of others • Organised PA opportunities
**Emotion**	• Feelings around PA maintenance • Goals for PA maintenance • Remembering the past and accepting the now • Other health conditions
**Behavioural Regulation**	• Keeping track and checking in on PA • Habits, routine and memory • Planning • Adapting PA, flexibility and resilience • Symptom management • Adapting PA in response to symptom level

Key: PA: physical activity; TDF: Theoretical Domains Framework

Four distinct PA patterns were constructed from participants experiences of maintaining PA since they finished their PMP: “consistently active”, “Initially consistently active post-PMP but then inconsistently active”, “inconsistently active since PMP” and “Same or a reduction in PA level since PMP”. The “consistently active” pattern includes participants who were doing a higher amount of weekly PA than before the PMP every week or nearly week at the time of the interview. This pattern includes participants who had been consistently active since the PMP finished. It also includes participants who were initially inconsistent after the PMP but were now consistent. The “Initially consistently active post-PMP but then inconsistently active” pattern describes a group of participants that had a period of doing a higher amount of weekly PA than before the PMP consistently post programme but were inconsistent with their weekly level at the time of the interview. The “inconsistently active since PMP” pattern includes participants that had been inconsistent with their weekly PA level since the PMP. On their higher weeks they were doing more PA than before the PMP but had been unable to do this consistently. The “same or a reduction in PA level since the PMP” pattern describes a smaller group of participants who’s weekly PA level had not changed or had reduced since the PMP. They described a downward trajectory in PA level. We have highlighted below where subthemes were prominent for barriers and facilitators (≥50% of participants in pattern reported barriers and facilitators within that subtheme). The prominent subthemes for barriers and facilitators in each pattern are also displayed in Table 7. See supplementary material for frequencies of each subtheme within the patterns.

**Table 7:** Prominent subthemes for barriers and facilitators within each PA pattern.

PA pattern	Prominent subthemes for facilitators within pattern	Prominent subthemes for barriers within pattern	Prominent subthemes for barriers and facilitators within pattern
Consistently active (n=10)	• Feelings around maintenance • Beliefs about the Value of PA maintenance • Believing in ability to maintain PA • Goals for PA maintenance • Remembering the past and accepting the now • Keeping track and checking in • Habits, routine and memory • Symptom management • Adapting PA in response to symptom level • Encouragement, motivation and positive emotional responses • Location of PA	• Competing demands and life roles and responsibilities	
Initially consistently active post-PMP but then inconsistently active (n=5)	• Beliefs about the Value of PA maintenance • Goals for PA maintenance • Habits, routine and memory • Encouragement, motivation and positive emotional responses	• Believing in ability to maintain PA • Competing demands and life roles and responsibilities • Other health conditions • Influence of persistent musculoskeletal pain symptoms • Negative influence of others	• Feelings around PA maintenance
Inconsistent since PMP (n=6)	• Feelings around PA maintenance • Beliefs about the value of PA maintenance • Believing in ability to maintain PA • Goals for PA maintenance • Habits, routine and memory • Symptom management • Adapting PA in response to symptom level	• Competing demands and life roles and responsibilities • Other health conditions • Influence of persistent musculoskeletal pain symptoms • Location of PA	• Feelings around PA maintenance • Encouragement, motivation and positive emotional responses
Same or a reduction in PA level since PMP (n=3)	• Goals for PA maintenance	• Beliefs about the value of PA maintenance • Believing in ability to maintain PA • Influence of persistent musculoskeletal pain symptoms • Location of PA	

Abbreviations: n: number; PA: physical activity; PMP: pain management programme

#### Theme 1: Internal drivers for PA maintenance

Participants discussed many internal factors that represent a person with pain’s thoughts and feelings about maintaining PA. There were six subthemes:

##### Feelings around PA maintenance

Almost all participants described how feelings related to PA can be barriers or facilitators. This subtheme maps onto the TDF domain Emotion. Feelings are prominent facilitators for those in the “consistently active” pattern but were barriers or facilitators for those in the two inconsistently active patterns.

Enjoyment was a prominent facilitator for many and social factors often contributing to this (e.g., meeting people during PA). For some, a lack of enjoyment was a barrier. Fear about PA was common. For many patient participants, the PMP helped reduce or learn to manage fear. Understanding pain, better symptom control and experiencing benefits from PA reduced fear. Using strategies to manage fear, such as reframing pain, enabled a few participants to persevere, which benefitted their mental health. This strategy-based approach was mapped to the TDF domain Skills. Fear of symptoms worsening from not doing PA was a motivator for a few participants. For some, fear remained a prominent barrier, including fear of falling, hurting/injuring themselves or pain increasing. Fear can return or increase due to lack of symptom control, pain flare-ups or new symptoms.

Less common emotions that facilitated PA included feeling happy, confident and proud about being active. Feeling safe during PA was a facilitator for some patient participants which was helped by doing it with others. In contrast, frustration occurred when participants had to reduce PA when pain increased. Some patient participants used self-compassion strategies to help overcome negative feelings about PA; again, this was mapped to the Skills TDF domain.

##### Beliefs about the value of PA maintenance

Almost all participants held positive beliefs, which were prominent facilitators for both the “consistently active” and two inconsistently active patterns. This subtheme maps to the TDF domain Beliefs about Consequences. Knowledge of the benefits and safety of PA gained from PMPs helped shape positive beliefs for some participants, and this subtheme mapped to the TDF domain Knowledge.

The perceived benefits of maintaining PA included physical and functioning (e.g., improved fitness), psychological and mental (e.g., improved mood), better general health, pain and condition management (which can lead to reduced medication), better sleep and improved work performance. Some patients and PA partners had both positive and negative beliefs (e.g., believe it’s beneficial but may be harmful). A few, mostly in the “same or reduction in PA level since PMP” pattern, described only negative beliefs towards PA, including the perception that doing PA incorrectly or too much could be harmful or a lack of belief it will help their pain.

##### Believing in ability to maintain PA

Participants described mixed confidence with abilities to maintain PA and this maps to the TDF domain Beliefs about Capabilities. For many participants, high confidence was often a facilitator, prominent in the “consistently active” and “inconsistently active since PMP” patterns. Some patients indicated they lacked confidence, which was a barrier. A few participants’ confidence fluctuated depending on recent PA experiences (e.g., how their pain was during or after). Lacking confidence was a prominent barrier for those in the “initially consistently active post-PMP but then inconsistently active” and “same or reduction in PA level since PMP” patterns. Participants felt more confident if they were experiencing benefits and limited pain flare-ups, managing PA physically, making progress and were supported by others. They felt confident they could recover from PA lapses if they had previously recovered, had progressed to higher PA levels before or had strategies to progress (e.g., pacing). Reasons for low confidence included flare-ups or high pain during or after PA, being uncertain how to progress PA, or being unsure about doing it correctly or about their body adapting and managing long-term. A few patient participants were not confident in certain environments (e.g., in public where they are seen). Having knowledge about PA (e.g., what constitutes PA) helped to build confidence with maintenance, whereas limited knowledge reduced confidence (TDF domain: Knowledge).

##### Goals for PA maintenance

Almost all patient participants had PA goals, and they were prominent facilitators for all PA patterns (TDF domain: Goals). Goals typically covered important aspects of participants’ lives (e.g., being physically able enough to have a child) or a benefit they wanted (e.g., good mental health) from PA maintenance. Many described that it was useful having a goal to work towards and that achieving goals often led to positive feelings that further facilitated PA. In contrast, a few participants did not like goal setting as not achieving goals can lead to a feeling of failure which is demotivating. These feelings resulted in this subtheme also being mapped to the TDF domain Emotion.

##### Remembering the past and accepting the now

Participants’ memories of PA before their pain started had varying impact. For some, memories of higher previous PA levels led to dissatisfaction with current PA, which can negatively impact their mood and motivation. It can also lead to overexertion. Lower pre-pain levels of activity can lead to greater satisfaction with current PA. Some others felt motivated by remembering positive feelings (e.g., enjoyment). Acceptance that fluctuations in pain, physical abilities and PA are normal helped with implementation of pain management strategies (e.g., pacing), which facilitated PA for some. A few explained that acceptance of pain and current activity levels helped to overcome negative comparisons with previous PA, reducing its impact. It also enhanced confidence towards PA, leading to this subtheme being mapped to the TDF domain Beliefs about Capabilities. Positive relationships with past PA and acceptance of current PA led to this subtheme being prominent for facilitators for the “consistently active” PA pattern. A lack of acceptance led to fear around PA and reduced mood which negatively impacted PA maintenance for a few participants. The effect of memories of past PA and acceptance on feelings led to this subtheme being mapped to the TDF domain Emotion. This subtheme relates to participants’ identity and perceptions of their PA levels, resulting in it being mapped to the TDF domain Social/Professional Role and Identity.

##### Keeping track and checking in on PA

Many participants described how monitoring their PA and noticing progress (e.g., higher step count) can be motivating and confidence boosting and result in a sense of pride. These positive feelings reinforce PA maintenance and correspond to the TDF domain Reinforcement. Monitoring can also be a reminder for PA. Reflecting on their body’s response to PA (e.g., pain intensity) can help judge whether they are doing the right amount or intensity. Monitoring and reflection were prominent facilitators for the “consistently active” PA pattern. Noticing a lower amount of PA than they would like can act as a prompt to increase activity, acting as a motivator, or, as a few mentioned, it can be demotivating which can lower their mood and be a barrier. Participants used step counters, activity diaries, phone logs, measuring how much they can do before needing a rest, and goal achievement to monitor PA. The use of monitoring and reflection corresponds to the TDF domain Behavioural Regulation.

#### Theme 2: Fitting physical activity into life

In this theme, participants explain how PA interacts with features of their lives, including competing demands, habits, routines and other health conditions. It describes strategies to manage PA alongside these factors, including planning and adapting PA.

##### Competing demands and life roles and responsibilities

Many participants described how competing demands, including family commitments (e.g., caring for others), social events, studying, working, volunteering and management of other health conditions can limit time for PA or lead to not prioritising PA, forgetting PA, limited energy or higher pain that affects PA. Life events (e.g., bereavement) can cause PA lapses. Competing demands were prominent barriers for the “consistently active” and both inconsistently active PA patterns. In contrast, having a purpose for PA (e.g., walking the dog) can be a facilitator, as can life roles (e.g., an aunty doing PA with their niece/nephew). This subtheme maps onto the TDF domains Environmental Context and Resources, Social Influences and Social/Professional Role and Identity as competing demands and life roles and responsibilities are features of a person’s environment, relate to professional and social roles and are influenced by others.

##### Habits, routine and memory

Forming habits and routines for PA are prominent facilitators of maintenance for those in the “consistently active” and both inconsistently active PA patterns, positively impacting many participants. Forming habits and routines regulates PA maintenance, resulting in this subtheme mapping to the TDF domain Behavioural Regulation. When participants had formed habits, PA required less mental effort and they relied less on memory. Some participants deliberately formed habits (e.g., by setting reminders until it became habitual). For some others it happened unconsciously. Continually acting on habits reinforces PA maintenance by strengthening the habit, resulting in this subtheme mapping to the TDF domain Reinforcement. Habit formation was reported to be hindered by strong competing habits, negative thoughts and feelings about PA (e.g., fear) and neurodivergent conditions (e.g., autism spectrum disorder). Some participants described having daily routines that PA was a part of or specific PA routines (e.g., specific exercise routine). Participants with a lack of daily routine highlighted this can affect motivation and consistency with PA. Remembering PA was challenging and acted as a barrier for some patient participants. Some patients used strategies to help with remembering PA, including reminders or prompts on their phones or activity watches, or putting it in their diaries. The challenges with remembering PA, as well as habit and routine formation leading to less required mental effort to maintain PA align with the TDF domain Memory, Attention and Decision Processes.

##### Planning PA

Some participants planned what PA they are going to do and when they are going to do it and described how planning could facilitate PA maintenance. This intent aligns with the TDF domain Intentions. The use of planning as a maintenance strategy aligns with the domain Behavioural Regulation. Having set days to do PA, supported by joining organised PA programmes, was helpful. Planning PA into their weeks was habitual for some. For some others, challenges with planning PA include neurodivergent disorders, symptom variability, competing demands, and avoidance of a feeling of failure from not achieving plans. While planning was important for some participants, it was not prominent for any of the PA patterns.

##### Other health conditions

Acute and long-term health conditions can be barrier to PA maintenance for many participants. They were prominent barriers for participants in both inconsistently active PA patterns. Acute health conditions can be temporary barriers and cause PA lapses. Chronic conditions (e.g., migraine) can be ongoing barriers or lead to PA lapses when flared up. These conditions reduce a person’s physical and mental capacity to do PA, resulting in this subtheme being mapped to the TDF domain Skills. They also affected some participants beliefs that they could do PA, resulting in this subtheme being mapped to the domain Beliefs about Capabilities. Many participants described a bidirectional relationship with pain and mental health; poor mental health can be a barrier to PA, but PA can lift mood. Due to this relationship with a person’s mood, this subtheme has been mapped to the TDF domain Emotion.

##### Adapting PA, flexibility and resilience

Being able to adapt and be flexible about their PA when facing competing demands, other health conditions, adverse weather or if they are unable to do it in their usual environments is an important facilitator of PA maintenance for some. However, this subtheme was not prominent for any PA pattern. The participants explained how being able to adapt the type, duration or intensity of PA are key to maintenance (e.g., gentle home-based PA instead of walking). Adapting PA is a skill aimed at regulating PA maintenance and, therefore, this subtheme has been mapped to the TDF domains Skills and Behavioural Regulation.

#### Theme 3: Symptoms and symptom management

This theme describes the negative impact that symptoms, particularly pain, fatigue and deconditioning, can have on PA maintenance. It explains how limited control over symptoms can be a barrier but the ability to manage these symptoms can be an important facilitator of PA maintenance. Adapting PA to symptom level helps consistency and limits period of inactivity when symptoms are higher.

##### Influence of persistent musculoskeletal pain symptoms

General higher pain and fatigue, and associated deconditioning, can make PA challenging for nearly all patient participants as it can affect their physical ability and activity tolerance. This resulted in this subtheme being mapped to the TDF domain Skills. Higher symptoms can also reduce confidence with PA maintenance (TDF domain: Beliefs about Capabilities). Consistent PA was particularly challenging for participants if it exacerbated their pain as this can make them avoidant of PA. Variable and unpredictable symptoms can lead to inconsistency, particularly if symptoms dictate PA. This can make it hard to plan and form routines. Many participants described having limited control of their symptoms in these situations. Flare-ups of pain can cause lapses or relapses and reduce motivation and confidence, leading to recurrence of fear. Higher pain and fatigue were prominent barriers for those in both inconsistently active and the “same or a reduction in PA level since the PMP” pattern.

##### Symptom management

Many participants described how improved and maintained symptom control is an important facilitator of PA maintenance, often using strategies they learnt on the PMP. Good symptom management was a prominent facilitator for the “consistently active” and “inconsistently active since PMP” patterns. Using pain management strategies to regulate PA maintenance aligns with the TDF domains Skills and Behavioural Regulation. Pacing was the most common strategy used to manage pain and, when used effectively, helped participants build confidence regarding PA. Other strategies included mindfulness, relaxation, distraction techniques and flare-up/setback plans. Using strategies to manage fear (e.g., mindfulness) can lead to physical changes (e.g., gait), which can reduce pain during PA. In contrast, a lack of effective pain management is a barrier to PA, as described in the previous section. For example, not pacing can lead to increases in pain and a subsequent need to rest. A few participants had further surgery since the PMP which negatively impacted consistent symptom control.

##### Adapting PA to symptom level

Many participants described how being able to adapt the intensity, duration, type, or their goals for PA depending on their symptoms enabled PA maintenance even when it was more difficult, such as during a flare-up. This was prominent for those in the “consistently active” and “inconsistently active since PMP” patterns. This subtheme maps on to the TDF domains Skills and Behavioural Regulation as being able to adapt is a skill and is aimed at ensuring PA maintenance.

#### Theme 4: Social networks and influences

Other people can be important influences on PA maintenance. They can provide encouragement and other positive emotional responses and practical support which can facilitate maintenance. However, a reliance on support from others can be a barrier when those people are unavailable. Other people can also negatively influence PA if they are unsupportive. All the subthemes below map on to the TDF domain Social Influences as they relate to interpersonal processes**.**

##### Encouragement, motivation and positive emotional responses

Nearly all participants in the “consistently active” and both inconsistently active patterns explained how encouragement from others facilitated PA maintenance by providing motivation, accountability, confidence and reduced fear regarding PA. This support came from family, friends, PA partners, PMP peers or HCPs. PA partners increased enjoyment of PA and enabled participants to push themselves more and feel safer, which was particularly important for those with fear of PA. Some participants that relied on PA partners described a vulnerability when their PA partner was unavailable as they could feel demotivated or more anxious about doing PA without them, which was a prominent barrier for those in the “inconsistently active since PMP” pattern. Feedback on progress helped to validate it, aiding motivation. Ongoing communication with their support network was key to ensuring that needs were understood. Ongoing support from PMP peers was also important when maintained long-term but broke down shortly after the programme for some participants. A few patient participants described how they felt encouraged by role models such as seeing their peers do PA, sport on TV or social media.

##### Practical support

Many participants described how family and friends can help them to implement pain management strategies, which subsequently helps PA maintenance. The few that had ongoing HCP support described how it helped them maintain confidence with problem-solving support, monitoring and progressing PA, and referrals to accessing community PA opportunities, including social prescribing. Some benefited from ongoing peer support to share ideas, advice and learning about maintaining PA, including signposting to community opportunities. Social media recommendations can also help with PA maintenance, however, patients advised caution with regards to their appropriateness. Some patient participants also described a vulnerability towards PA consistency if they are reliant on their PA partners for practical support such as help with balance, particularly if they have a history of falls. Participants across all PA patterns highlighted practical support as facilitators and/or barriers. However, it was not prominent for any of the patterns.

##### Negative influence of others

Some participants explained how a lack of understanding about their needs, negative beliefs about pain and PA, or feeling pressured by family can be barriers to PA maintenance. Perceived judgement, shame or stigma, linked to a lack of understanding, were barriers for a few participants. These negative influences were prominent barriers for the “initially consistently active post-PMP but then inconsistently active” pattern.

#### Theme 5: Environmental influences

Almost all participants explained how the availability, suitability and accessibility of PA can be barriers or facilitators to PA uptake and maintenance post-PMP. There are also financial influences for some participants, which are mostly barriers. They also discussed the availability and suitability of PA groups/classes and influence of the instructors on whether they engaged with the these.

##### Location of PA

Having easily accessible PA places was an important facilitator for many participants and was a prominent facilitator for the “consistently active” pattern. Having safe environments such as a park or the beach nearby often made PA more enjoyable, providing mental benefits. Some participants highlighted that their living environment is important; comfort and space can facilitate PA whereas clutter and noise can be barriers. Accessibility issues, including access to buildings and swimming pools, which were not adapted for people with mobility problems or did not have suitable parking were barriers for some participants. A few participants that did not drive and did not have places near their homes explained how they were unable to access places for PA as they had no accessible public transport options. Some find travelling tiring which negatively impacts their PA. Some participants described how gyms and public swimming pools are often unsuitable for people with persistent pain as they can be intimidating, the equipment can be hard to use with limited strength, pools are too cold or are not setup for exercises other than swimming. These issues were prominent barriers for the “inconsistently active since PMP” and “same or a reduction in PA level since the PMP” PA patterns. This subtheme maps to the TDF domain Environmental Context and Resources as it relates to features of the participants’ environment.

##### Financial influences

Financial influences on PA maintenance were described by some participants and were mostly barriers. They included the cost of accessing PA facilities and classes/groups, and the cost of transport to get to places for PA. A few participants stated that government financial support is not enough to support PA. Free or reduced costs for PA can be a facilitator. Financial influences were mapped onto the TDF domain Environmental Context and Resources as they relate to a person’s environment. A few participants described how making a financial commitment can facilitate PA, as long as they do not overdo the activity. Financial commitments resulted in this subtheme also being mapped onto the TDF domain Intentions. Although important influences for some participants, this subtheme was not prominent for any of the PA patterns.

##### Organised PA opportunities

Some patient participants joined PA groups or classes in their communities to increase PA, explore other types of PA (e.g., gym) and for social contact, which they believed would increase their confidence. Some highlighted that many groups/classes are not suitable for people with pain and disability and so they avoid them. The availability of suitable groups/classes was described as a “postcode lottery”. This subtheme maps to the TDF domain Environmental Context and Resources as the availability of groups/classes relates to the participants’ environment. Class instructors’ knowledge about persistent pain can be a barrier or facilitator. The influence of class instructors resulted in this subtheme being mapped to the TDF domain Social Influences. While this subtheme was important for some participants, it was not prominent for any of the patterns.

##### Weather

Some participants described how the weather can affect PA maintenance (TDF domain: Environmental Context and Resources). Bad weather can be a barrier as pain can be higher on colder days. PA is also less enjoyable during bad weather. This can make it harder to be consistent during winter. In contrast, good weather can facilitate PA. This subtheme was not prominent for any of the PA patterns.

### **Interactions between subthemes and TDF domains**

Our analysis generated five interactions between subthemes that we consider significant and that show how TDF domains can interact. Specifically, the subtheme “encouragement, motivation and positive emotional responses from others” can positively influence “belief in ability to maintain PA”. The TDF domains mapped to these subthemes indicate that Social Influences can positively influence Beliefs about Capabilities. The subtheme “Practical support” positively influenced “Feelings around PA maintenance”, indicating that Social Influences can positively influence the TDF domain Emotion. The subtheme “Believing in the value of PA” positively influenced “Goals for PA maintenance” and “Feelings around PA maintenance”, shows that the TDF domain Beliefs about Consequences can positively influence Goals and Emotion. Lastly, the subtheme “Competing demands and life roles and responsibilities” positively and negatively influenced “Habit, routine and memory”. These subthemes all map onto multiple TDF domains, highlighting that Social/Professional Role and Identity, Environmental Context and Resources, and Social Influences can positively and negatively influence Reinforcement, Memory, Attention and Decision Processes, and Behavioural Regulation.

Table 8 summarises these interactions and provides examples of the interactions between barriers/facilitators within subthemes. See supplementary material for all the interactions generated between subthemes and the number of codes, data fragments and statements of significance within each interaction.

**Table 8:** Interactions between subthemes, directions of interactions and examples of barrier/facilitators interactions within subthemes.

**Subtheme interaction**^**(TDF domains – see key)**^	**Direction of interaction**	**Example of how barriers/facilitators within subthemes can interact with each other**	**Participant quotes for interactions**
**Encouragement, motivation & positive emotional responses from others**^**(8)**^ influencing **Belief in ability to maintain PA**^**(2)**^	Positive	Doing PA in a group and receiving social support through this helps build confidence for PA	“So, if the group members in the PMP can actually still keep in contact, maybe do some sort of regular activities together, I definitely feel like that will boost their confidence to try things on their own in the future.” (HCP 4)
**Practical support**^**(8)**^ influencing **Feelings around PA maintenance**^**(9)**^	Positive	Doing PA with someone else facilitates PA by reducing fear of falling	“My daughter, she's got a bit of a weird job. You never know, she never knows what time she finishes, and she never knows what time she starts, but if she finishes early, she might come out with me. So she'll support me while we take the dog for a little walk. So I look forward to that, because I know I'm safe if she's supporting me.” (Patient 22)
**Competing demands and life roles and responsibilities**^**(1,7,8)**^ influencing **Habit, routine & memory**^**(4,6,10)**^	Positive and negative	Continually prioritising PA over competing demands facilitates habit formation (positive)Previously had PA routine that has been broken by competing demands (negative)	“So it was very good for me, and there was definitely a habit, and not so much now […] Because life, literally, because, you know, I'd rather meet your girlfriend for a coffee, or, you know, I've got paperwork I need to do, or work or whatever. So it's just, and then, you know. And then there's always the in the background, how much is that going to hurt afterwards now?” (Patient 21)
**Believing in value of PA**^**(3)**^ influencing **goals for PA maintenance**^**(5)**^	Positive	Believing they will benefit from maintaining PA leads to setting a goal of being able to go on holiday and manage the holiday.	**Participant:** “We would like to, I think, really go, be able to go on holiday…”**Researcher:** “And does, does that goal help you with your physical activity at all?”**Participant: “**Yes, because I know that it will improve me and get me better. Yeah. I mean, even if I have the rest before I know that we're going to have a busy day, and perhaps we might have one busy day and one quiet day, but that's doable as well.” (Patient 17)
**Believing in value of PA**^**(3)**^ influencing **Feelings around PA maintenance**^**(9)**^	Positive	Perception that they are benefitting from PA leads to reduction of fear of PA	“I know I need to move, however, what means whether to do 10 min a day or an hour a day. For me, I know that's what I need to do, because I don't want to go in that dark hole again of pain, holding myself stiff, depression, not going out, stopping everything.” (Patient 13)

**Key:** PA: physical activity

TDF domains corresponding numbers:

(1) Social/Professional Role and Identity

(2) Beliefs about Capabilities

(3) Beliefs about Consequences

(4) Reinforcement

(5) Goals

(6) Memory, Attention and Decision Processes

(7) Environmental Context and Resources

(8) Social Influences

(9) Emotion

(10) Behavioural Regulation

## Discussion

Maintaining PA after PMPs is complex and challenging for many people with persistent musculoskeletal pain. We explored the barriers and facilitators to maintaining PA following PMPs and identified PA patterns following PMPs. Barriers and facilitators varied widely across participants, highlighting the importance of individual context. Five themes were generated that described this complex experience, with findings mapped onto 13 TDF domains. The interactions identified between subthemes reflected the interdependent nature of potential barriers and facilitators. The four PA patterns which were constructed highlight the variation in PA maintenance trajectories.

Many of the barriers and facilitators described by participants were similar to those reported in previous studies concerning PA participation for people with musculoskeletal conditions or persistent pain.^19^, ^30^, ^31^ We have built on these findings by generating knowledge about how these barriers and facilitators specifically influence PA maintenance. For example, previous studies have described the negative impact of fear on PA participation;^19^, ^30^ we describe how fear can remain a barrier after PMPs, but it’s impact can be lessened if people can implement management strategies. Furthermore, negative feelings towards PA can return after a PMP even if these feelings reduced during the programme. Another example is that most participants believed in the benefits of PA post-PMP which can facilitate PA maintenance, but these beliefs can become a barrier if they do not experience benefits. This shows how barriers and facilitators are dynamic and can change over time, indicating a need for long-term support to overcome new barriers or to manage returning barriers.

Multiple subthemes mapped to the TDF domains Skills and Behavioural Regulation. This contrasts with our previous systematic review which did not focus specifically on PA maintenance, where these domains were coded to less than others.^19^ These domains may be particularly prominent for PA maintenance because our participants had attended PMPs that introduce pain management strategies and encourage PA adaptability and flexibility. These strategies were more commonly a facilitator among those in the “consistently active” pattern. Furthermore, PA habits and routines were common facilitators for participants that were consistent with PA but were not commonly reported in previous studies not specific to maintenance,^19^, ^30^, ^31^ suggesting an important role specifically during PA maintenance. PA self-monitoring and reflection, used mostly by those consistent with PA, may also be more useful specifically during PA maintenance as they have also had limited mention in previous studies not specific to maintenance. Overall, our findings suggest that some barriers and facilitators are more prominent during PA maintenance and highlights the need for an intervention that specifically targets this phase.

Tailoring interventions is important for this population due to the varied trajectories of PA maintenance, evidenced by the four novel PA patterns constructed in this study. The different PA patterns suggest that PA consistency can fluctuate and there is a susceptibility to lapses and relapses (e.g., people can be consistent post-PMP and then become inconsistent and vice versa). A PA maintenance intervention could offer tailoring dependent on these patterns; for example, our results indicate that those in the inconsistently active patterns may benefit from more support targeting symptom management. Our results also indicate that those in the “consistently active” pattern may require a less intensive intervention as they have less barriers. Furthermore, if someone’s PA pattern changes, the intervention components could be adapted to align with this.

All the subthemes that were prominent for facilitators for the two inconsistently active patterns were also prominent for the “consistently active” pattern. However, multiple other subthemes were prominent for facilitators for the “consistently active” pattern than others. This highlights that the facilitators are consistent across the patterns, but the “consistently active” group are making use of multiple others as well. In addition, more subthemes were prominent for barriers for the other patterns than the “consistently active” group. This suggests that there are more potential barriers for those finding PA maintenance more challenging. This suggests that an intervention that is tailored to different patterns is warranted.

A key difference between the PA patterns is that patients in the two inconsistently active and the “same or a reduction in PA level since PMP” patterns appeared more negatively affected by their persistent pain symptoms and their other health issues than patients in the “consistently active” pattern, whose PA was facilitated by symptom management strategies and adapting their PA. Believing in the value of PA was commonly a facilitator among patients in the “consistently active” and both inconsistently active PA patterns as they perceived the benefits of PA. This suggests that while positive beliefs may support people doing PA, it is not enough without other facilitators to support consistency. Having goals related to PA was common across all groups, likely because PMPs often promote goal setting. However, while described as helpful by most participants, being present across all PA patterns suggests it doesn’t affect PA maintenance alone. Competing demands was a common barrier among all patterns, indicating the challenges managing these external influences.

Many factors that were described as facilitators by some were not used by other patients; for example, PA habits and/or routines were described as very important for some patients, but many had not developed these facilitators. Some patients may struggle to form habits even with support, such as we learnt from some patients with neurodivergent conditions. This highlights the individuality of barriers and facilitators and how interventions need to account for the likelihood that even with support, some people will not be able to benefit from certain strategies. Tailoring the intervention can account for this.

Exploring interactions between different barriers and facilitators, and subsequently TDF domains, supports intervention development by generating insights into which may be important factors to prioritise targeting in the intervention. For example, we found that the TDF domain Social Influences positively influences Beliefs about Capabilities and Emotion and, therefore, it could be beneficial to prioritise targeting Social Influences. This is particularly useful when using the Behaviour Change Wheel (BCW), a framework that systematically guides intervention development.^17^ When using the BCW, TDF domains map on to the Capability, Opportunity, Motivation-Behaviour (COM-B) model, which explains how capability, opportunity and motivation interact to generate behaviour. Therefore, identifying which TDF domains to prioritise enables selection of the most appropriate COM-B constructs. Following this, the next step of the BCW framework is to select the intervention types that correspond with that COM-B construct. Being able to confidently prioritise the most influential constructs will likely lead to development of a more efficient intervention with less components.

Many of the barriers and facilitators discussed by the participants could be addressed by an intervention delivered in healthcare settings and/or a self-management intervention. However, some prominent factors, including the availability of appropriate, accessible and affordable PA opportunities for people with persistent pain are influenced by public health and environmental factors. Healthcare/self-management interventions can support patients to do PA that is possible for them, however, many will be limited by the opportunities available to them, even if the intervention is tailored to their environment, which is important for long-term maintenance.

### Strengths and limitations

A strength is that we generated rich qualitative data from different stakeholders from multiple sites representing different PMP structures and geographical variation across the UK. Our analysis consisted of multiple layers using an inductive approach to generate rich insights from the data, followed by a deductive approach using an established behaviour change theoretical framework.^25^ This will enable the results to inform intervention development in later phases of the PAMPER study. We have constructed novel PA patterns and generated unique insights into how different barriers and facilitators can influence each other which will enhance the theoretical foundation of future intervention development. We satisfied our sampling criteria and had good representation in other patient characteristics (besides ethnicity). We purposefully included participants that self-identified as having difficulty with consistent PA maintenance, a limitation of previous studies.^19^ Lastly, the study was supported by PPI throughout.

This study has limitations: all PA partners were romantic partners, and different insights may have been generated if there were more varied relationships. Most of the patient participants were of White ethnicity; more variation may have led to different barriers/facilitators or contextual differences in how the barriers and facilitators present. This also means the findings may not be transferable to people from other ethnic communities. The HCPs were at times speculating about potential barriers and facilitators as they do not often see participants post-PMP due to service design or working patterns. The PA patterns were generated from a small sample and the number of participants varied between patterns.

Our work has highlighted a possible method for generating insights into how barriers and facilitators, and subsequently TDF domains, interact. Many more potential links between barriers and facilitators were identified but these were not common and it was difficult to decide whether to include them. Future work would benefit from developing criteria on what constitutes an interaction between barriers and facilitators in qualitative studies (e.g., via consensus methods). A larger dataset may support this by enabling generation of more insights.

## Conclusion

We generated five themes representing the barriers and facilitators to PA maintenance following PMPs among people with persistent musculoskeletal pain. These themes are explained by multiple subthemes that map on to 13 out of 14 TDF domains. We generated unique insights on interactions between subthemes and subsequently TDF domains. These findings will inform the selection of appropriate intervention targets when developing a PA maintenance intervention. We also constructed four PA patterns describing different trajectories of PA maintenance. These patterns can inform tailoring of the intervention to ensure that patients receive the most appropriate intervention at the right time to facilitate PA maintenance.

## CRediT authorship contribution statement

**GB:** Conceptualisation, methodology, formal analysis, investigation, writing – original draft, writing – review and editing, visualisation, project administration, funding acquisition. **DD:** methodology, formal analysis, writing – review and editing, supervision. **LB:** conceptualisation, methodology, writing – review and editing, supervision. **MU:** conceptualisation, methodology, formal analysis, writing – review and editing, supervision.

## Disclosures

Gregory Booth is funded by the NIHR Doctoral Fellowship (NIHR303240). The views expressed are those of the author(s) and not necessarily those of the NIHR or the Department of Health and Social Care.

## Declaration of Competing Interest

The authors declare no competing interests.

## Footnotes

Appendix A Supplementary data associated with this article can be found in the online version at doi:10.1016/j.jpain.2026.106235.
