# Supplementary material for: Maintaining physical activity after pain management programmes for people with persistent musculoskeletal pain: A qualitative exploration of barriers, facilitators and activity patterns
Source: J Pain. 2026 May;42:None. doi: 10.1016/j.jpain.2026.106235 (PMC13168399; doi:10.1016/j.jpain.2026.106235)
Supplement: Supplementary file 1 — Supplementary material [file mmc1.docx]

Supplementary file – topic guide iteration

**Topic guide PAMPER phase 1 (patient and physical activity partner)**

**Warm up questions and experience of physical activity since the pain management programme**

*First, I’ll ask some questions about your current physical activity and how you have found it since the pain management programme.*

Can you tell me what physical activity you do?

Where do you do your physical activity?

*Prompt: such as at home, leisure centre, a class?*

When we first spoke on the phone, you told me you were [consistently active, inconsistently active, inactive], can you tell me more about how much physical activity you do and when you do it?

How do you feel about keeping up physical activity long-term?

How have you found doing physical activity since finishing the pain management programme at [organisation]?

*Prompt: have you done as much as you wanted to? If yes, why? If not, why not?*

Is the physical activity that you do now different to when you were doing the pain management programme?

*Prompt: if so, what is different and why?*

**Barriers, facilitators, and intervention components**

*I am now going to ask you about what helps and hinders keeping up your physical activity.*

What makes keeping up your physical activity difficult? *(open question and discuss answers in more detail before going on to ask more specific questions below)*

*Prompt: is there anything else you can think of that makes keeping up physical activity difficult for you?*

What helps you to keep up your physical activity? *(open question and discuss answers in more detail before going on to ask more specific questions below)*

*Prompt: is there anything else you can think of that helps you to keep up your physical activity?*

*Planning, and intentions/routine/competing demands/burden*

*Next, I’ll ask you about your goals and plans for physical activity and how it fits into your life.*

Do you have a goal of how much physical activity you want to keep doing or what you want to gain from it?

*Prompts:*

- *If yes, why? What is your goal?*
- *If not, why not?*

How important are these goals for you?

How often do you achieve these goals?

*Prompts:*

- ***If achieves goals:*** *how confident are you that you can continue to meet your goal? What influences your confidence?*
- ***If does not achieve goals:*** *how confident are you that you can achieve these goals in the future? What influences your confidence?*
- ***If sometimes achieve goals:*** *how confident are you that can achieve these goals more often? What influences your confidence?*

Do the goals change?

*Prompts:*

- *If yes, why? What changes about them?*
- *If not, why not?*

Are you planning to keep up regular physical activity long-term?

*Prompt: Why do you plan (or not plan) to keep it up?*

Are your plans to do physical activity different now or the same compared to when you did the pain management programme?

How much do you plan your physical activity?

*Prompt: do you have a structure to when and how you do your physical activity? If so, does it help? If so, why? If not, why not?*

How does (or would) physical activity affect and fit into your lifestyle?

*Prompt: Are there any other roles or responsibilities that you have that affect your physical activity? For example, work or family. If so, how do these affect your physical activity?*

Do you ever have to decide between doing physical activity or something else?

*Prompt: For example, other pain management strategies, working or seeing family. If so, what do you tend to prioritise? How do you make these decisions?*

How do you find doing physical activity along with your other pain management strategies?

*Prompt: do you find it difficult to use multiple pain management strategies. If yes, why? If not, why not? How do you prioritise them?*

Can you think of anything that might help you or other people prioritise their physical activity or be able to keep it up despite other roles and responsibilities?

Have you ever identified as a physically active person?

*Follow-up: If yes, how important is this for your long-term physical activity? If not, do you think this impacts your physical activity? Has this changed since the pain management programme?*

*Beliefs about capabilities/confidence related to long-term PA*

*I’m now going to ask you about your confidence for keeping active long-term.*

How confident are you that you can keep up physical activity long-term?

How confident are you that you can get back to your desired level of physical activity if you have a dip in your activity or period of not being active?

What influences your confidence to keep up physical activity or get back to it if you have a dip?

How important do you think confidence is when keeping up physical activity? Why?

How confident are you that physical activity will be beneficial for you in the long-term?

*Prompt: If confident, why? If not, why not?*

How can people build their confidence to keep physically active, or keep confident to stay active?

*Feelings/experiences about maintaining PA*

*Now I’d like to ask you about your feelings around physical activity.*

How do you feel when doing physical activity?

*Prompt: do you feel anything good (like enjoyment) or bad (like fear).*

*Follow-up: Have these feelings changed since you first started doing physical activity with pain? If so, how?*

*Follow-up: Do these feelings influence how much physical activity you do?*

*Follow-up: How do you manage these feelings?*

*Problem solving, memory regarding PA and monitoring PA*

*I’m now going to ask you about times when it is difficult to keep up physical activity and about remembering to do it.*

In situations when it is more difficult to keep up your physical activity, how much of your physical activity are you able to do?

*Prompt: The same, less? Why?*

Are these situations the same or different to when you were increasing your physical activity during the pain management programme?

What do you do, if anything, to help you keep up your physical activity in these situations?

Can you think of anything that might help people to keep up their physical activity in these situations, or recover from periods where they are less active?

Do you ever have problems remembering to do your physical activity? If yes, why? If not, why not?

Is there anything you do to help you remember or that reminds you to do your physical activity?

*Prompt: for example, alarms on your phone.*

*Prompt: are these helpful? Do they work to remind you?*

*Prompt: can you think of anything else that could remind people to do their activity?*

How much has your physical activity become a habit?

If habitual:

- How much does this help you remember to do physical activity?
- Does this affect how much effort it takes to be active?
- Did you deliberately try and form the habit, or did it just happen?
- What did you do to try and make it a habit?

If not habitual:

- Do you think it would be helpful to be more of a habit?
- What do you think could make it more of a habit?

*Prompt: what do you think about adding it to an existing habit so that it becomes related to that habit? (e.g., taking a walk after a meal).*

Do you use anything to monitor or keep track of how much physical activity you do?

*Prompt: for example, a diary or fitness watch?*

*Follow-up: if so, does it help? If so, why? If not, why not?*

*Knowledge and beliefs about keeping up physical activity*

*I’ll now ask you about your knowledge and thoughts about keeping up physical activity.*

Do you know the benefits of keeping up physical activity?

*Prompt: Could you tell me what the most important ones are for you, that most motivate you to be active?*

*Prompt: do you think there are any downsides to keeping up physical activity? If so, what do you think they are?*

Do you think there are any benefits or downsides to not keeping up physical activity?

How important do you think consistency is when keeping up physical activity?

*Prompt: What do you think the impact of dips in physical activity level or periods of being inactive might be? Why?*

Do you know how to keep up physical activity?

*Prompt: do you know what to do, how much to do, where you can do it and how you can do it?*

What is important for people to know about keeping up physical activity with persistent musculoskeletal pain?

How could people with persistent pain be supported to improve and maintain their knowledge about physical activity?

*Social influences and communication*

*I’m now going to ask you about any support you receive for physical activity.*

Can you think of anything anyone has said or done that made you more motivated to be physically active?

Can you think of anything anyone has said or done that made you less motivated to be active?

**If individual interview:**

Do you have anybody that gives you support to keep up your physical activity?

*Prompt such as family, friends, healthcare professionals?*

*Prompt: if so, how do they support you? How important is this support?*

*Prompt: if not – how beneficial do you feel support from other people could be? Who do you feel that support would be beneficial from? And what could that look like?*

If at all, how do you talk to other people about your physical activity?

*Prompt: what do you talk to them about or tell them?*

How important is communicating with other people about keeping up your activity?

*If important, why? If not, why not?*

If they get support for PA: how did you go about getting this support for your physical activity? How could others get support for keeping up physical activity for themselves?

**If dyadic interview:**

In what ways does [name of PA partner] support you to keep up physical activity?

*Prompt if PA partner is someone they do PA with: What happens if [name of PA partner] is not available?*

How important is this support to you for keeping up your physical activity?

Do you have any other people who support you to keep up physical activity?

*Prompt: if so, in what ways do they support you?*

*Prompt: how important is this support?*

How did you go about getting this support for your physical activity? How could others get support for keeping up physical activity for themselves?

**Intervention characteristics**

You have already told me what might help people with certain aspects of keeping up physical activity, such as [list intervention characteristics so far]. As you know, we are going to design a support package to help people keep up physical activity after a pain management programme. I am now going to ask you some more specific questions about how people with your condition could be supported to keep up their physical activity.

Can you think of anything else that might help people, like yourself, to keep up physical activity after a pain management programme?

What could the support package include?

Who would be best to provide the support?

How could the support work?

*Prompts:*

- *Would you prefer to receive support face-to-face, via telephone or video call, digital media or a different way? Why?*
- *You mentioned earlier that X may be helpful, how could that look in a support package?*
- *Would you prefer to get support in a group or on your own? Why?*
- *How would the content of the support be delivered to you? For example, digitally, such as website or app, in a workbook?*

Where would be best for the support to work?

*Prompts:*

- *At home? How and why?*
- *Community centre? How and why?*
- *Healthcare centre? How and why?*

When do you think you would need the support package?

How much support do you think you would need to help you keep up physical activity? Would this vary or always be the same? Why?

If the support package could be personalised, how could that work? for example, could it be tailored to symptoms, certain barriers, or physical activity levels?

**Ending the interview**

*We are now at the end of the interview. Is there anything you’d like to add to anything we have discussed or anything new you’d like to mention?*

*Thank you for participating in the interview. I will now stop the recording.*

Supplementary file – mapping subtheme interactions to TDF, showing number of codes, data fragments and quotes expressing significance of interaction, with examples of codes and direction of influence

| **TDF domains** | **Links between subthemes** | **Direction of influence (positive or negative)** | **Link code(s)** |
| --- | --- | --- | --- |
| **Knowledge influencing Emotion**  **Codes in link: 4**  **Data fragments: 4**  **Links between subthemes: 1** | Believing in value of PA influencing Feelings around PA maintenance  4 codes/data fragments | Positive (beliefs influencing emotion)  Negative (knowledge influencing emotion) | Lack of knowledge about how to progress PA can lead to return of negative thoughts, particularly if they try to progress and flare-up |
|  |  |  | Lack of knowledge about they can and can't do leads to worry about PA |
|  |  |  | No longer fearful of PA as experiencing benefits of PA |
|  |  |  | PA facilitated by better understanding of pain, reduced fear and more positive attitude |
| **Social/Professional Role & Identity influencing Reinforcement**  **Codes in link: 5**  **Data fragments: 5 (1 significant)**  **Links between subthemes: 1** | **Competing demands influencing Habit, routine & memory**  **5 codes/data fragments (1 significant)** | Positive (prioritising PA over competing demands helps habit)  Negative (competing demands affecting memory, routine and habit) | Competing demands can lead to forgetting to do PA |
|  |  |  | Continually prioritising PA facilitates habit formation |
|  |  |  | Feels PA being habitual and routine would be helpful - previous had PA routine that has been broken by competing demands and thoughts about pain afterwards |
|  |  |  | Has daily routine for some forms of PA but walking depends on competing demands and PA partner availability |
|  |  |  | Working zero hour contracts so unable to build consistent PA routine |
| **Social/Professional Role & Identity influencing Behavioural Regulation**  **Codes in link: 9**  **Data fragments: 9 (1 significant)**  **Links between subthemes: 3** | Competing demands influencing Habit, routine & memory  5 codes/data fragments | Positive (prioritising PA over competing demands helps habit)  Negative (competing demands affecting memory, routine and habit) | Competing demands can lead to forgetting to do PA |
|  |  |  | Continually prioritising PA facilitates habit formation |
|  |  |  | Feels PA being habitual and routine would be helpful - previous had PA routine that has been broken by competing demands and thoughts about pain afterwards |
|  |  |  | Has daily routine for some forms of PA but walking depends on competing demands and PA partner availability |
|  |  |  | Working zero hour contracts so unable to build consistent PA routine |
|  | Competing demands influencing Symptom management  1 code/data fragment | Negative | Life event that leads to forgetting to do PA could lead to people overdoing it trying to catch up |
|  | Remembering past & accepting now influencing Symptom management  3 codes/ data fragments (1 significant) | Positive (acceptance = better symptom management)  Negative (lack of acceptance = poor symptom management) | Acceptance of age and physical limitations important for allowing self to pace PA |
|  |  |  | Acceptance that they can't do same amount of PA as before pain facilitates PA maintenance by allowing pacing |
|  |  |  | Lack of acceptance and self-compassion can result in patients finding it hard to pace PA |
| **Social/Professional Role & Identity influencing Skills**  **Codes in link: 5**  **Data fragments: 5 (1 significant)**  **Links between subthemes: 3** | Competing demands influencing Symptom management  1 code/data fragment | Negative | Life event that leads to forgetting to do PA could lead to people overdoing it trying to catch up |
|  | Competing demands influencing Persistent musculoskeletal pain symptoms  1 code/data fragment | Negative | Working leads to exhaustion which makes PA hard |
|  | Remembering past & accepting now influencing Symptom management  3 codes/data fragments (1 significant) | Positive (acceptance = better symptom management)  Negative (lack of acceptance = poor symptom management) | Acceptance of age and physical limitations important for allowing self to pace PA |
|  |  |  | Acceptance that they can't do same amount of PA as before pain facilitates PA maintenance by allowing pacing |
|  |  |  | Lack of acceptance and self-compassion can result in patients finding it hard to pace PA |
| **Social/Professional Role & Identity influencing Beliefs about Capabilities**  **Codes in link: 1**  **Data fragments: 1**  **Links between subthemes: 1** | Competing demands influencing Persistent musculoskeletal pain Symptoms  1 code/ data fragment | Negative | Working leads to exhaustion which makes PA hard |
| **Social/Professional Role & Identity influencing Memory, Attention & Decision Processes**  **Codes in link: 5**  **Date fragments: 5**  **Links between subthemes: 1** | Competing demands influencing Habit, routine & memory  5 codes/data fragments | Positive (prioritising PA over competing demands helps habit)  Negative (competing demands affecting memory, routine and habit) | Competing demands can lead to forgetting to do PA |
|  |  |  | Continually prioritising PA facilitates habit formation |
|  |  |  | Feels PA being habitual and routine would be helpful - previous had PA routine that has been broken by competing demands and thoughts about pain afterwards |
|  |  |  | Has daily routine for some forms of PA but walking depends on competing demands and PA partner availability |
|  |  |  | Working zero hour contracts so unable to build consistent PA routine |
| **Intentions influencing Skills**  **Codes in link: 1**  **Date fragments: 1**  **Links between subthemes: 1** | Financial influences influencing Symptom management  1 code/ data fragment | Negative | Paying gym memberships can lead to overdoing PA due to obligation to make most of membership |
| **Intentions influencing Behavioural Regulation**  **Codes in link: 1**  **Date fragments: 1**  **Links between subthemes: 1** | Financial influences influencing Symptom management  1 code/data fragment | Negative | Paying gym memberships can lead to overdoing PA due to obligation to make most of membership |
| **Reinforcement influencing Beliefs about Capabilities**  **Codes in link: 2**  **Date fragments: 2 (2 very significant)**  **Links between subthemes: 1** | Keeping track & checking in influencing Belief in ability to maintain PA  2 codes/data fragments (2 significant) | Positive | Feels positive about PA maintenance due to knowledge gained on PMP and seeing progress from PA post-PMP |
|  |  |  | Plans for PA have increased since PMP as building up PA and recognises what their body can do |
| **Environmental Context and Resources influencing Behavioural Regulation**  **Codes in link: 9**  **Data fragments: 9 (4 significant)**  **Links between subthemes: 4** | **Competing demands influencing Habit, routine & memory**  **5 codes/data fragments (3 significant)** | Positive (prioritising PA over competing demands helps habit)  Negative (competing demands affecting memory, routine and habit) | Competing demands can lead to forgetting to do PA |
|  |  |  | Continually prioritising PA facilitates habit formation |
|  |  |  | Feels PA being habitual and routine would be helpful - previous had PA routine that has been broken by competing demands and thoughts about pain afterwards |
|  |  |  | Has daily routine for some forms of PA but walking depends on competing demands and PA partner availability |
|  |  |  | Working zero hour contracts so unable to build consistent PA routine |
|  | Competing demands influencing Symptom management  1 code/data fragment | Negative | Life event that leads to forgetting to do PA could lead to people overdoing it trying to catch up |
|  | Financial influences influencing Symptom management  1 code/data fragment (1 significant) | Negative | Paying gym memberships can lead to overdoing PA due to obligation to make most of membership |
|  | Location influencing Habit, routine & memory  2 codes/data fragments | Negative | Negative environments can trigger inactivity habitually |
|  |  |  | Unsatisfactory living environment leads to lack of routine which affects PA consistency |
| **Environmental Context and Resources influencing Skills**  **Codes in link: 3**  **Data fragments: 3 (2 significant)**  **Links between subthemes: 3** | Competing demands influencing Symptom management  1 code/data fragment | Negative | Life event that leads to forgetting to do PA could lead to people overdoing it trying to catch up |
|  | Competing demands influencing Persistent musculoskeletal pain symptoms  1 code/data fragment (1 significant) | Negative | Working leads to exhaustion which makes PA hard |
|  | Financial influences influencing Symptom management  1 code/data fragment (1 significant) | Negative | Paying gym memberships can lead to overdoing PA due to obligation to make most of membership |
| **Environmental Context and Resources influencing Emotion**  **Codes in link: 1**  **Data fragments: 1**  **Links between subthemes: 1** | Location influencing Feeling around PA maintenance  1 code/data fragment | Negative | Anxious about going out to do PA but unsuitable home environment |
| **Environmental Context and Resources influencing Beliefs about Capabilities**  **Codes in link: 2**  **Data fragments: 2**  **Links between subthemes: 2** | Competing demands influencing Persistent musculoskeletal pain symptoms  1 code/data fragment | Negative | Working leads to exhaustion which makes PA hard |
|  | Location of PA and Beliefs in ability to maintain PA  1 code/data fragment | Negative | Unsatisfactory living environment affects confidence with PA maintenance |
| **Environmental Context and Resources influencing Beliefs about Consequences**  **Codes in link: 2**  **Data fragments: 3 (2 significant)**  **Links between subthemes: 1** | Location of PA influencing Beliefs about value of PA  2 codes/3 data fragments (2 significant) | Positive | Being near local park and beach facilitates PA due as provides mental benefits |
|  |  |  | Having enjoyable places to walk and activities to do provides mental benefits of PA |
| **Environmental Context and Resources influencing Memory, Attention & Decision Processes**  **Codes in link: 5**  **Data fragments: 5 (3 significant)**  **Links between subthemes: 1** | **Competing demands influencing Habit, routine & memory**  **5 codes/data fragments (3 significant)** | Positive (prioritising PA over competing demands helps habit)  Negative (competing demands affecting memory, routine and habit) | Competing demands can lead to forgetting to do PA |
|  |  |  | Continually prioritising PA facilitates habit formation |
|  |  |  | Feels PA being habitual and routine would be helpful - previous had PA routine that has been broken by competing demands and thoughts about pain afterwards |
|  |  |  | Has daily routine for some forms of PA but walking depends on competing demands and PA partner availability |
|  |  |  | Working zero hour contracts so unable to build consistent PA routine |
| **Environmental Context and Resources influencing Reinforcement**  **Codes in link: 7**  **Data fragments: 7 (4 significant)**  **Links between subthemes**: **2** | **Competing demands influencing Habit, routine & memory**  **5 codes/data fragments (3 significant)** | Positive (prioritising PA over competing demands helps habit)  Negative (competing demands affecting memory, routine and habit) | Competing demands can lead to forgetting to do PA |
|  |  |  | Continually prioritising PA facilitates habit formation |
|  |  |  | Feels PA being habitual and routine would be helpful - previous had PA routine that has been broken by competing demands and thoughts about pain afterwards |
|  |  |  | Has daily routine for some forms of PA but walking depends on competing demands and PA partner availability |
|  |  |  | Working zero hour contracts so unable to build consistent PA routine |
|  | Location influencing Habit, routine & memory  2 codes/data fragments (1 significant) | Negative | Negative environments can trigger inactivity habitually |
|  |  |  | Unsatisfactory living environment leads to lack of routine which affects PA consistency |
| **Social Influences influencing Emotion**  **Codes in link: 10**  **Data fragments: 10 (6 significant)**  **Links between subthemes: 3** | Encouragement, motivation & positive emotional responses from others influences Feeling towards PA maintenance  3 codes/data fragments (1 significant) | Positive | Doing PA with someone else makes it more enjoyable |
|  |  |  | Friend meeting them at the pool helps enjoy PA more |
|  |  |  | Ongoing physiotherapy support helps people know they are doing PA correctly which reduces fear |
|  | Encouragement, motivation & positive emotional support from others influencing Other health conditions  1 code/data fragment (1 significant) | Positive | Social element of PA can help with mood and overall wellbeing |
|  | **Practical support influencing Feelings around PA maintenance**  **6 codes/data fragments (4 significant)** | Positive | Feels safe when doing PA with daughter but daughter not often available |
|  |  |  | Ongoing peer and significant other support can help reduce fear of PA when fear occurs |
|  |  |  | Ongoing physio support post-PMP helps to manage fear of PA |
|  |  |  | Only does PA if has someone to do it with due to fear of falling |
|  |  |  | PA partners can help people feel safe to do PA |
|  |  |  | PA reduces anxiety about doing PA outside and going for a walk |
| **Social Influences influencing Beliefs about Capabilities**  **Codes in link: 11**  **Data Fragments: 11 (7 significant)**  **Links between subthemes: 5** | **Encouragement, motivation & positive emotional responses from others influencing Belief in ability to maintain PA**  **7 codes/data fragments (4 significant)** | Positive | Doing PA in a group and receiving social support through this helps build confidence for PA |
|  |  |  | Doing PA with PMP peers could help boost confidence with PA |
|  |  |  | Lack of confidence to do PA alone |
|  |  |  | Ongoing physio support post-PMP helps to maintain confidence with PA |
|  |  |  | Peer support can help confidence to maintain PA |
|  |  |  | Physios helped increase belief in this capabilities |
|  |  |  | Support system helps confidence for PA which then effects motivation |
|  | Practical support influencing Believing in ability to maintain PA  1 code/data fragment (1 significant) | Positive | Ongoing physio support post-PMP helps to maintain confidence with PA - link between HCP support and confidence |
|  | Encouragement, motivation & positive emotional support from others influencing Other health conditions  1 code/data fragment (1 significant) | Positive | Social element of PA can help with mood and overall wellbeing |
|  | Negative influence of others influencing persistent musculoskeletal pain symptoms  1 code/data fragment (1 significant) | Negative | Unexpected events planned by family increase pain and make PA hard |
|  | Competing demands influencing persistent musculoskeletal pain symptoms  1 code/data fragment | Negative | Working leads to exhaustion which makes PA hard |
| **Social Influences influencing Beliefs about Consequences**  **Codes in link: 3**  **Data fragments: 3 (2 significant)**  **Links between subthemes: 2** | Negative influence of others influencing Beliefs about value of PA maintenance  2 codes/data fragments (1 significant) | Negative | Negative beliefs can be influenced by social influences |
|  |  |  | Negative beliefs from family or HCPs affect uptake and maintenance |
|  | Practical support influencing Belief in the value of PA which influences Goals for PA maintenance  1 code/data fragment (1 significant) | Positive | Goal and intentions are to keep going to the gym as HCPs told them the importance of movement |
| **Social Influences influencing Behavioural Regulation**  **Codes in link: 11**  **Data fragments: 11 (3 significant)**  **Links between subthemes: 6** | Practical support influencing Habit, routine & memory  2 codes/data fragments | Positive | Formed routine for PA as told it would be beneficial by PMP team |
|  |  |  | Has daily routine for some forms of PA but walking depends on competing demands and PA partner availability |
|  | Practical support influencing Adapting PA, flexibility & resilience  1 code/data fragment | Positive | Good communication between person with pain and family members facilitates problem-solving |
|  | Encouragement, motivation & positive emotional responses from other influencing Symptom management  1 code/data fragment (1 significant) | Positive | Highly important that those they live with are supportive and understanding of limitations to help them pace appropriately |
|  | Encouragement, motivation & positive emotional responses from others influencing Habit, routine & memory  1 code/data fragment | Positive | Habit formation facilitated by consistent practice and social support to provide ongoing motivation |
|  | **Competing demands influencing Habit, routine & memory**  **5 codes/data fragment (2 significant)** | Positive (prioritising PA over competing demands helps habit)  Negative (competing demands affecting memory, routine and habit) | Competing demands can lead to forgetting to do PA |
|  |  |  | Continually prioritising PA facilitates habit formation |
|  |  |  | Feels PA being habitual and routine would be helpful - previous had PA routine that has been broken by competing demands and thoughts about pain afterwards |
|  |  |  | Has daily routine for some forms of PA but walking depends on competing demands and PA partner availability |
|  |  |  | Working zero hour contracts so unable to build consistent PA routine |
|  | Competing demands influencing Symptom management  1 code/data fragment | Negative | Life event that leads to forgetting to do PA could lead to people overdoing it trying to catch up |
| **Social Influences influencing Skills (Physical)**  **Codes in link: 1**  **Data fragments: 1 (1 significant)**  **Links between subthemes: 1** | Negative influence of others influencing persistent musculoskeletal pain symptoms  1 code/data fragment (1 significant) | Negative | Unexpected events planned by family increase pain and make PA hard |
| **Social Influences influencing Skills (Psychological)**  **Codes in link: 4**  **Data fragments: 4 (2 significant)**  **Links between subthemes: 4** | Encouragement, motivation & positive emotional responses from other influencing Symptom management  1 code/data fragment (1 significant) | Positive | Highly important that those they live with are supportive and understanding of limitations to help them pace appropriately |
|  | Practical support influencing Adapting PA, flexibility & resilience  1 code/data fragment | Positive | Good communication between person with pain and family members facilitates problem-solving |
|  | Competing demands influencing Symptom management  1 code/data fragment | Negative | Life event that leads to forgetting to do PA could lead to people overdoing it trying to catch up |
|  | Competing demands influencing persistent musculoskeletal pain symptoms  1 code/data fragment (1 significant) | Negative | Working leads to exhaustion which makes PA hard |
| **Social Influences influencing Reinforcement**  **Codes in link: 6**  **Data fragments: 6 (1 significant)**  **Links between subthemes: 2** | Encouragement, motivation & positive emotional responses from others influencing Habit, routine & memory  1 code/data fragment | Positive | Habit formation facilitated by consistent practice and social support to provide ongoing motivation |
|  | **Competing demands influencing Habit, routine & memory**  **5 codes/data fragments (1 significant)** | Positive (prioritising PA over competing demands helps habit)  Negative (competing demands affecting memory, routine and habit) | Competing demands can lead to forgetting to do PA |
|  |  |  | Continually prioritising PA facilitates habit formation |
|  |  |  | Feels PA being habitual and routine would be helpful - previous had PA routine that has been broken by competing demands and thoughts about pain afterwards |
|  |  |  | Has daily routine for some forms of PA but walking depends on competing demands and PA partner availability |
|  |  |  | Working zero hour contracts so unable to build consistent PA routine |
| **Social Influences influencing Memory, Attention & Decision Processes**  **Codes in link: 6**  **Data fragments: 6 (1 significant)**  **Links between subthemes: 2** | Encouragement, motivation & positive emotional responses from others influencing Habit, routine & memory  1 code/data fragment | Positive | Habit formation facilitated by consistent practice and social support to provide ongoing motivation |
|  | **Competing demands influencing Habit, routine & memory**  **5 codes/data fragments (1 significant)** | Positive (prioritising PA over competing demands helps habit)  Negative (competing demands affecting memory, routine and habit) | Competing demands can lead to forgetting to do PA |
|  |  |  | Continually prioritising PA facilitates habit formation |
|  |  |  | Feels PA being habitual and routine would be helpful - previous had PA routine that has been broken by competing demands and thoughts about pain afterwards |
|  |  |  | Has daily routine for some forms of PA but walking depends on competing demands and PA partner availability |
|  |  |  | Working zero hour contracts so unable to build consistent PA routine |
| **Emotion influencing Reinforcement**  **Codes in link: 2**  **Data fragments: 2 (1 significant)**  **Links between subthemes: 1** | Feelings around PA influencing Habit, routine & memory  2 codes/data fragments (1 significant) | Negative | Feels PA being habitual and routine would be helpful - previous had PA routine that has been broken by competing demands and thoughts about pain afterwards |
|  |  |  | Habit formation limited by fear of hurting self through PA |
| **Emotion influencing Behavioural Regulation**  **Codes in link: 7**  **Data fragments: 7 (2 significant)**  **Links between subthemes: 3** | Feelings around PA influencing Habit, routine & memory  2 codes/data fragments | Negative | Feels PA being habitual and routine would be helpful - previous had PA routine that has been broken by competing demands and thoughts about pain afterwards |
|  |  |  | Habit formation limited by fear of hurting self through PA |
|  | Feelings around PA influencing Symptom management  2 codes/data fragments (1 significant) | Positive (better feeling control = better symptom management)  Negative (Not controlling feelings = poor symptom management) | Lack of acceptance and self-compassion can result in patients finding it hard to pace PA |
|  |  |  | More self-compassion leads to them allowing themself to pace |
|  | Remembering past & accepting now influencing Symptom management  3 codes/data fragments (1 significant) | Positive (acceptance = better symptom management)  Negative (lack of acceptance = poor symptom management) | Acceptance of age and physical limitations important for allowing self to pace PA |
|  |  |  | Acceptance that they can't do same amount of PA as before pain facilitates PA maintenance by allowing pacing |
|  |  |  | Lack of acceptance and self-compassion can result in patients finding it hard to pace PA |
| **Emotion influencing Skills**  **Codes in link: 6**  **Data fragments: 6 (3 significant)**  **Links between subthemes: 3** | Feelings towards PA influencing Symptom management  2 codes/data fragments (1 significant) | Positive (better feeling control = better symptom management)  Negative (Not controlling feelings = poor symptom management) | Lack of acceptance and self-compassion can result in patients finding it hard to pace PA |
|  |  |  | More self-compassion leads to them allowing themself to pace |
|  | Feelings towards PA influencing persistent musculoskeletal pain symptoms  1 code/data fragment (1 significant) | Positive | Fear still present but manages it by reframing pain and using mindfulness - has led to better control of symptoms and more PA |
|  | Remembering past & accepting now influencing Symptom management  3 codes/data fragments (1 significant) | Positive (acceptance = better symptom management)  Negative (lack of acceptance = poor symptom management) | Acceptance of age and physical limitations important for allowing self to pace PA |
|  |  |  | Acceptance that they can't do same amount of PA as before pain facilitates PA maintenance by allowing pacing |
|  |  |  | Lack of acceptance and self-compassion can result in patients finding it hard to pace PA |
| **Emotion influencing Emotion**  **Codes in link: 2**  **Data fragments: 2**  **Links between subthemes: 2** | Feelings towards PA influencing Other health conditions  1 code/data fragment | Positive | Better control of pain and less stress about pain positively effects mental health |
|  | Remembering past & accepting now influencing Other health conditions  1 code/data fragment | Negative | Lack of acceptance of pain can lead to ongoing fear and is related to mental health |
| **Emotion influencing Beliefs about Consequences**  **Codes in link: 1**  **Data fragments: 1**  **Links between subthemes: 1** | Feelings towards PA influencing persistent musculoskeletal pain symptoms  1 code/data fragment | Positive | Fear still present but manages it by reframing pain and using mindfulness - has led to better control of symptoms and more PA |
| **Skills influencing Emotion**  **Codes in link: 5**  **Data fragments: 5 (4 significant)**  **Links between subthemes: 3** | Symptom management influencing Feelings around PA maintenance  1 code/data fragment (1 significant) | Positive | Not overdoing PA by pacing makes PA more enjoyable |
|  | Symptom management influencing Other health conditions  1 code/data fragment (1 significant) | Positive | Better control of pain and less stress about pain positively effects mental health |
|  | Persistent musculoskeletal pain symptoms influencing Feelings around PA maintenance  3 codes/data fragments (2 significant) | Negative | Fear can return even if reduced during PMP particularly during and post flare-up |
|  |  |  | High pain makes PA difficult as unable to do usual volume or intensity - leads to conflicting feelings - annoyed not done as much but happy has done something |
|  |  |  | New injuries or symptoms can lead to recurrence of kinesiophobia - link between flare-ups and emotions |
| **Skills influencing Skills**  **Codes in link: 3**  **Data fragments: 3**  **Links between subthemes: 2** | Feelings around PA maintenance influencing Symptom management  2 codes/data fragments | Positive (better feeling control = better symptom management)  Negative (Not controlling feelings = poor symptom management) | Lack of acceptance and self-compassion can result in patients finding it hard to pace PA |
|  |  |  | More self-compassion leads to them allowing themself to pace |
|  | Feelings around PA maintenance influencing Persistent musculoskeletal pain symptoms  1 code/data fragment | Positive | Fear still present but manages it by reframing pain and using mindfulness - has led to better control of symptoms and more PA |
| **Skills influencing Behavioural Regulation**  **Codes in link: 2**  **Data fragments: 2 (1 significant)**  **Links between subthemes: 1** | Feelings around PA maintenance influencing Symptom management  2 codes/data fragments (1 significant) | Positive (better feeling control = better symptom management)  Negative (Not controlling feelings = poor symptom management) | Lack of acceptance and self-compassion can result in patients finding it hard to pace PA |
|  |  |  | More self-compassion leads to them allowing themself to pace |
| **Skills (physical) influencing Memory, Attention & Decision Processes**  **Codes in link: 2**  **Data fragments: 2 (1 significant)**  **Links between subthemes: 2** | Other health conditions influencing Habits, routine & memory  1 code/data fragment | Negative | Cognition can affect memory for PA - neurodiversity as an example |
|  | Persistent musculoskeletal pain symptoms influencing Habit, routine & memory  1 code/data fragment (1 significant) | Negative | Hard to remember PA due to lack of routine and brain fog and PA not being habitual - link between routine, brain fog and habit |
| **Skills influencing Goals**  **Codes in link: 1**  **Data fragments: 1**  **Links between subthemes: 1** | Symptom management influencing goals  1 code/data fragment | Negative | Pacing PA beneficial - does not set targets so that they don't overdo it |
| **Skills influencing Beliefs about Capabilities**  **Codes in link: 3**  **Data fragments: 3 (1 significant)**  **Links between subthemes: 2** | Symptom management influencing Believing in ability to maintain PA  1 code data fragment | Positive | Confident they can recover from lapses using pacing |
|  | Persistent musculoskeletal pain symptoms influencing Believing in ability to maintain PA  2 codes/data fragments (1 significant) | Negative | Lacks confidence in PA maintenance due to high pain |
|  |  |  | Pain flare-ups led to reduced confidence and subsequently motivation |
| **Skills influencing Beliefs about Consequences**  **Codes in link: 1**  **Data fragments: 1**  **Links between subthemes: 1** | Feelings around PA maintenance influencing Persistent musculoskeletal pain symptoms  1 code/data fragment | Positive | Fear still present but manages it by reframing pain and using mindfulness - has led to better control of symptoms and more PA |
| **Skills influencing Environmental Context and Resources**  **Codes in link: 1**  **Data fragments: 1**  **Links between subthemes: 1** | Adapting PA, flexibility and resilience influencing impact of Competing demands & life roles responsibilities  1 code/data fragment | Positive | Being flexible with PA to work it around other commitments helpful |
| **Skills influencing Social/Professional Role & Identity**  **Codes in link: 1**  **Data fragments: 1**  **Links between subthemes: 1** | Adapting PA, flexibility and resilience influencing impact of Competing demands & life roles responsibilities  1 code/data fragment | Positive | Being flexible with PA to work it around other commitments helpful |
| **Skills influencing Social Influences**  **Codes in link: 1**  **Data fragments: 1**  **Links between subthemes: 1** | Adapting PA, flexibility and resilience influencing impact of Competing demands & life roles responsibilities  1 code/data fragment | Positive | Being flexible with PA to work it around other commitments helpful |
| **Behavioural Regulation influencing Environmental Context and Resources**  **Codes in link: 1**  **Data fragments: 1**  **Links between subthemes: 1** | Adapting PA, flexibility and resilience influencing impact of Competing demands & life roles responsibilities  1 code/data fragment | Positive | Being flexible with PA to work it around other commitments helpful |
| **Behavioural Regulation influencing Social/Professional Role & Identity**  **Codes in link: 1**  **Data fragments: 1**  **Links between subthemes: 1** | Adapting PA, flexibility and resilience influencing impact of Competing demands & life roles responsibilities  1 code/data fragment | Positive | Being flexible with PA to work it around other commitments helpful |
| **Behavioural Regulation influencing Social Influences**  **Data fragments: 1**  **Codes in link: 1**  **Links between subthemes: 1** | Adapting PA, flexibility and resilience influencing impact of Competing demands & life roles responsibilities  1 code/data fragment | Positive | Being flexible with PA to work it around other commitments helpful |
| **Behavioural Regulation influencing Beliefs about Capabilities**  **Codes in link: 5**  **Data fragments: 5 (2 significant)**  **Links between subthemes: 4** | Habit, routine & memory influencing Belief in ability to maintain PA  1 code/data fragment | Positive | Has PA routine that is gradually building up - confident with routine as has led to only a few manageable flare-ups |
|  | Habit, routine & memory influencing Persistent musculoskeletal pain symptoms  1 code/data fragment | Positive | Well-paced structure and routine for PA helps manage pain associated with PA |
|  | Keeping track & checking in influencing Belief in ability to maintain PA  2 codes/data fragments (2 significant) | Positive | Feels positive about PA maintenance due to knowledge gained on PMP and seeing progress from PA post-PMP |
|  |  |  | Plans for PA have increased since PMP as building up PA and recognises what their body can do |
|  | Symptom management influencing Believing in ability to maintain PA  **1 code/data fragment** | Positive | Confident they can recover from lapses using pacing |
| **Behavioural Regulation influencing Emotion**  **Codes in link: 2**  **Data fragments: 2 (1 significant)**  **Links between subthemes: 2** | Symptom management influencing Feelings around PA maintenance  **1 code/data fragment (1 significant)** | Positive | Not overdoing PA by pacing makes PA more enjoyable |
|  | Symptom management influencing Other health conditions  **1 code/data fragment** | Positive | Better control of pain and less stress about pain positively effects mental health |
| **Behavioural Regulation influencing Goals**  **Codes in link: 1**  **Data fragments: 1**  **Links between subthemes: 1** | Symptom management influencing goals  **1 code/data fragment** | Negative | Pacing PA beneficial - does not set targets so that they don't overdo it |
| **Behavioural Regulation influencing Skills**  **Codes in link: 1**  **Data fragments: 1**  **Links between subthemes: 1** | Habit, routine & memory influencing Persistent musculoskeletal pain symptoms  **1 code/data fragment** | Positive | Well-paced structure and routine for PA helps manage pain associated with PA |
| **Beliefs about Consequences influencing Beliefs about Capabilities**  **Codes in link: 1**  **Data fragments: 1**  **Links between subthemes: 1** | Believing in value of PA influencing Beliefs about ability to maintain PA  1 code/data fragment | Positive | Very confident they can maintain PA due to the benefits they get from it and as it most beneficial pain management they have tried |
| **Beliefs about Consequences influencing Emotion**  **Codes in link: 4**  **Data fragments: 4 (2 significant)**  **Links between subthemes: 1** | **Believing in value of PA influencing Feelings around PA maintenance**  **4 codes/data fragments (2 significant)** | Positive (beliefs influencing emotion)  Negative (knowledge influencing emotion) | Lack of knowledge about how to progress PA can lead to return of negative thoughts, particularly if they try to progress and flare-up |
|  |  |  | Lack of knowledge about they can and can't do leads to worry about PA |
|  |  |  | No longer fearful of PA as experiencing benefits of PA |
|  |  |  | PA facilitated by better understanding of pain, reduced fear and more positive attitude |
| **Beliefs about Consequences influencing Goals**  **Codes in link: 5**  **Data fragments: 5 (1 significant)**  **Links between subthemes: 1** | **Believing in value of PA influencing goals for PA maintenance**  **5 codes/data fragments (1 significant)** | Positive | Does PA as aims to look and feel good |
|  |  |  | Goal is to be able to go on holiday and believes maintaining physical activity will help them achieve that goal |
|  |  |  | Has a goal related to outcome of PA due to the perceived benefits of doing PA |
|  |  |  | High motivation for PA as want to get back to doing things they enjoy but are no longer doing |
|  |  |  | Positive beliefs about PA impacting life goals |
| **Beliefs about Consequences influencing Environmental Context and Resources**  **Codes in link: 3**  **Data fragments: 3**  **Links between subthemes: 1** | Believing in value of PA influencing prioritising PA (influences decision-making around competing demands)  3 codes/data fragments | Positive | Prioritises PA due to benefits on health |
|  |  |  | Prioritises PA due to the mental health benefits |
|  |  |  | Prioritises PA due to the perceived benefits |
| **Beliefs about Consequences influencing Social/Professional Role & Identity**  **Codes in link: 3**  **Data fragments: 3**  **Links between subthemes: 1** | Believing in value of PA influencing prioritising PA (influences decision-making around competing demands)  3 codes/data fragments | Positive | Prioritises PA due to benefits on health |
|  |  |  | Prioritises PA due to the mental health benefits |
|  |  |  | Prioritises PA due to the perceived benefits |
| **Beliefs about Consequences influencing Social Influences**  **Codes in link: 3**  **Data fragments: 3 (1 significant)**  **Links between subthemes: 1** | Believing in value of PA influencing prioritising PA (influences decision-making around competing demands)  3 codes/data fragments (1 significant) | Positive | Prioritises PA due to benefits on health |
|  |  |  | Prioritises PA due to the mental health benefits |
|  |  |  | Prioritises PA due to the perceived benefits |
| **Beliefs about Capabilities influencing Emotion**  **Codes in link: 4**  **Data fragments: 4 (2 significant**  **Links between subthemes: 2** | Persistent musculoskeletal pain symptoms influencing Feelings around PA maintenance  3 codes/data fragments (2 significant) | Negative | Fear can return even if reduced during PMP particularly during and post flare-up |
|  |  |  | High pain makes PA difficult as unable to do usual volume or intensity - leads to conflicting feelings - annoyed not done as much but happy has done something |
|  |  |  | New injuries or symptoms can lead to recurrence of kinesiophobia - link between flare-ups and emotions |
|  | Remembering past & accepting now influencing Other health conditions  1 code/data fragment | Negative | Lack of acceptance of pain can lead to ongoing fear and is related to mental health |
| **Beliefs about Capabilities influencing Memory, Attention & Decision Processes**  **Codes in link: 1**  **Data fragments: 1**  **Links between subthemes: 1** | Persistent musculoskeletal pain symptoms influencing Habit, routine & memory  1 code/data fragment | Negative | Hard to remember PA due to lack of routine and brain fog and PA not being habitual - link between routine, brain fog and habit |
| **Beliefs about Capabilities influencing Skills**  **Codes in link: 3**  **Data fragments: 3 (2 significant)**  **Links between subthemes: 1** | Remembering past & accepting now influencing Symptom management  3 codes/data fragments (2 significant) | Positive (acceptance = better symptom management)  Negative (lack of acceptance = poor symptom management) | Acceptance of age and physical limitations important for allowing self to pace PA |
|  |  |  | Acceptance that they can't do same amount of PA as before pain facilitates PA maintenance by allowing pacing |
|  |  |  | Lack of acceptance and self-compassion can result in patients finding it hard to pace PA |
| **Beliefs about Capabilities influencing Behavioural Regulation**  **Codes in link: 3**  **Data fragments: 3 (2 significant)**  **Links between subthemes: 1** | Remembering past & accepting now influencing Symptom management  3 codes/ data fragments (2 significant) | Positive (acceptance = better symptom management)  Negative (lack of acceptance = poor symptom management) | Acceptance of age and physical limitations important for allowing self to pace PA |
|  |  |  | Acceptance that they can't do same amount of PA as before pain facilitates PA maintenance by allowing pacing |
|  |  |  | Lack of acceptance and self-compassion can result in patients finding it hard to pace PA |

Supplementary file - Frequency of subthemes within each PA pattern

| **Subtheme**  (TDF domains) | **Consistently active (n=10)** | **Initially consistently active post-PMP but then inconsistently active (n=5)** | **Inconsistent since PMP (n=6)** | **Same or a reduction in PA level since PMP (n=3)** |
| --- | --- | --- | --- | --- |
| **Theme:** Internal drivers for physical activity maintenance | | | | |
| **Feelings around PA maintenance**  (Emotion, Skills) | Facilitator **x8 (80%)**  Barrier x0  Barrier & facilitator **x2 (20%)** | Facilitator **x1 (20%)**  Barrier x0  Barrier & facilitator **x4 (80%)** | Facilitator **x3 (50%)**  Barrier x0  Barrier & facilitator **x3 (50%)** | Facilitator **x1 (33%)**  Barrier x0  Barrier & facilitator **x1 (33%)** |
| **Beliefs about the value of PA maintenance**  (Beliefs about Consequences, Knowledge) | Facilitator **x9 (90%)**  Barrier x0  Barrier & facilitator **x1 (10%)** | Facilitator **x3 (60%)**  Barrier x0  Barrier & facilitator **x2 (40%)** | Facilitator **x3 (50%)**  Barrier x0  Barrier & facilitator **x2 (33%)** | Facilitator x0  Barrier **x2 (67%)**  Barrier & facilitator **x1 (33%)** |
| **Believing in ability to maintain PA**  (Beliefs about Capabilities, Knowledge) | Facilitator **x5 (50%)**  Barrier **x1 (10%)**  Barrier & facilitator **x2(20%)** | Facilitator **x1 (20%)**  Barrier **x3 (60%)**  Barrier & facilitator x0 | Facilitator **x3 (50%)**  Barrier **x2 (33%)**  Barrier & facilitator x0 | Facilitator x0  Barrier **x2 (67%)**  Barrier & facilitator x0 |
| **Goals for PA maintenance**  (Goals, Emotion) | Facilitator **x9 (90%)**  Barrier x0  Barrier & facilitator x0 | Facilitator **x4 (80%)**  Barrier x0  Barrier & facilitator x0 | Facilitator **x5 (83%)**  Barrier **x1 (17%)**  Barrier & facilitator x0 | Facilitator **x2 (67%)**  Barrier x0  Barrier & facilitator x0 |
| **Remembering the past and accepting the now**  (Emotion, Social/Professional Role and Identity, Beliefs about Capabilities) | Facilitator **x6 (60%)**  Barrier **x1 (10%)**  Barrier & facilitator **x1 (10%)** | Facilitator x0  Barrier x0  Barrier & facilitator **x2 (40%)** | Facilitator **x2 (33%)**  Barrier **x2 (33%)**  Barrier & facilitator **x1 (17%)** | Facilitator x0  Barrier **x1 (33%)**  Barrier & facilitator x0 |
| **Keeping track and checking in on PA**  (Behavioural Regulation, Reinforcement) | Facilitator **x7 (70%)**  Barrier x0  Barrier & facilitator x0 | Facilitator **x1 (20%)**  Barrier x0  Barrier & facilitator x0 | Facilitator x0  Barrier x0  Barrier & facilitator x0 | Facilitator **x1 (33%)**  Barrier x0  Barrier & facilitator x0 |
| **Theme:** fitting physical activity into life | | | | |
| **Competing demands and life roles and responsibilities**  (Social Influences, Environmental Context and Resources, Social/Professional Role and Identity) | Facilitator x0  Barrier **x7 (70%)**  Barrier & facilitator x0 | Facilitator x0  Barrier **x5 (100%)**  Barrier & facilitator x0 | Facilitator x0  Barrier **x3 (50%)**  Barrier & facilitator x0 | Facilitator x0  Barrier **x1 (33%)**  Barrier & facilitator x0 |
| **Habits, routine and memory**  (Reinforcement, Behavioural Regulation, Memory, Attention and Decision Processes) | Facilitator **x9 (90%)**  Barrier x0  Barrier & facilitator **x1 (10%)** | Facilitator **x3 (60%)**  Barrier x0  Barrier & facilitator **x1 (20%)** | Facilitator **x5 (83%)**  Barrier x0  Barrier & facilitator x0 | Facilitator x0  Barrier **x1 (33%)**  Barrier & facilitator x0 |
| **Planning**  (Behavioural Regulation, Intention) | Facilitator **x4 (40%)**  Barrier x0  Barrier & facilitator x0 | Facilitator x0  Barrier x0  Barrier & facilitator x0 | Facilitator **x1 (17%)**  Barrier x0  Barrier & facilitator x0 | Facilitator **x1 (33%)**  Barrier x0  Barrier & facilitator x0 |
| **Other health conditions**  (Skills, Emotion, Beliefs about Capabilities) | Facilitator x0  Barrier **x2 (20%)**  Barrier & facilitator x0 | Facilitator x0  Barrier **x3 (60%)**  Barrier & facilitator x0 | Facilitator x0  Barrier **x4 (67%)**  Barrier & facilitator x0 | Facilitator x0  Barrier **x1 (33%)**  Barrier & facilitator x0 |
| **Adapting PA, flexibility and resilience**  (Skills, Behavioural Regulation) | Facilitator **x3 (30%)**  Barrier x0  Barrier & facilitator x0 | Facilitator **x2 (40%)**  Barrier x0  Barrier & facilitator x0 | Facilitator x0  Barrier x0  Barrier & facilitator x0 | Facilitator x0  Barrier x0  Barrier & facilitator x0 |
| **Theme:** Symptoms and symptom management | | | | |
| **Influence of persistent musculoskeletal pain symptoms**  (Skills, Belief about Capabilities) | Facilitator x0  Barrier **x4 (40%)**  Barrier & facilitator x0 | Facilitator x0  Barrier **x5 (100%)**  Barrier & facilitator x0 | Facilitator x0  Barrier **x6 (100%)**  Barrier & facilitator x0 | Facilitator x0  Barrier **x3 (100%)**  Barrier & facilitator x0 |
| **Symptom management**  (Skills, Behavioural Regulation) | Facilitator **x8 (80%)**  Barrier x0  Barrier & facilitator x0 | Facilitator x0  Barrier x0  Barrier & facilitator x0 | Facilitator **x3 (50%)**  Barrier x0  Barrier & facilitator x0 | Facilitator **x1 (33%)**  Barrier x0  Barrier & facilitator x0 |
| **Adapting PA in response to symptom level**  (Skills, Behavioural Regulation) | Facilitator **x7 (70%)**  Barrier x0  Barrier & facilitator x0 | Facilitator **x2 (40%)**  Barrier x0  Barrier & facilitator x0 | Facilitator **x3 (50%)**  Barrier x0  Barrier & facilitator x0 | Facilitator x0  Barrier x0  Barrier & facilitator x0 |
| **Theme:** Social networks and influences | | | | |
| **Encouragement, motivation and positive emotional responses**  (Social Influences) | Facilitator **x8 (80%)**  Barrier x0  Barrier & facilitator **x1 (10%)** | Facilitator **x3 (60%)**  Barrier x0  Barrier & facilitator x0 | Facilitator **x3 (50%)**  Barrier x0  Barrier & facilitator **x3 (50%)** | Facilitator **x1 (33%)**  Barrier x0  Barrier & facilitator **x1 (33%)** |
| **Practical support**  (Social Influences) | Facilitator **x4 (40%)**  Barrier x0  Barrier & facilitator **x2 (20%)** | Facilitator **x1 (20%)**  Barrier x0  Barrier & facilitator x0 | Facilitator **x2 (33%)**  Barrier x0  Barrier & facilitator **x1 (17%)** | Facilitator x0  Barrier x0  Barrier & facilitator **x1 (33%)** |
| **Negative influence of others**  (Social Influences) | Facilitator x0  Barrier **x1 (10%)**  Barrier & facilitator x0 | Facilitator x0  Barrier **x3 (60%)**  Barrier & facilitator x0 | Facilitator x0  Barrier **x2 (33%)**  Barrier & facilitator x0 | Facilitator x0  Barrier **x1 (33%)**  Barrier & facilitator x0 |
| **Theme:** Environmental influences | | | | |
| **Location of PA**  (Environmental Context and Resources) | Facilitator **x5 (50%)**  Barrier x0  Barrier & facilitator **x2 (20%)** | Facilitator **x2 (40%)**  Barrier x0  Barrier & facilitator **x1 (20%)** | Facilitator **x2 (33%)**  Barrier **x3 (50%)**  Barrier & facilitator **x1 (17%)** | Facilitator x0  Barrier **x3 (100%)**  Barrier & facilitator x0 |
| **Financial influences**  (Environmental Context and Resources, Intention) | Facilitator **x1 (10%)**  Barrier **x1 (10%)**  Barrier & facilitator x0 | Facilitator x0  Barrier **x1 (20%)**  Barrier & facilitator **x1 (20%)** | Facilitator **x1 (17%)**  Barrier **x2 (33%)**  Barrier & facilitator **x1 (17%)** | Facilitator x0  Barrier **x1 (33%)**  Barrier & facilitator x0 |
| **Organised PA opportunities**  (Environmental Context and Resources, Social Influences) | Facilitator **x1 (10%)**  Barrier **x2 (20%)**  Barrier & facilitator x0 | Facilitator x0  Barrier **x2 (40%)**  Barrier & facilitator x0 | Facilitator **x1 (17%)**  Barrier **x1 (17%)**  Barrier & facilitator **x1 (17%)** | Facilitator **x1 (33%)**  Barrier x0  Barrier & facilitator x0 |
| **Weather**  (Environmental Context and Resources) | Facilitator x0  Barrier **x3 (30%)**  Barrier & facilitator x0 | Facilitator x0  Barrier **x1 (20%)**  Barrier & facilitator x0 | Facilitator x0  Barrier **x2 (33%)**  Barrier & facilitator **x1 (17%)** | Facilitator x0  Barrier x0  Barrier & facilitator x0 |
| PA pattern definitions:   - Consistently active – Currently doing a higher amount of weekly PA than before the PMP every week or nearly every week - Initially consistently active post-PMP but then inconsistently active – Currently doing a higher amount of weekly PA than before the PMP some weeks but was initially consistently more active following the PMP. - Inconsistently active – Been doing a higher amount of weekly PA than before the PMP inconsistently since the PMP - Same or a reduction in PA level since PMP – No change or a reduction in weekly PA level since the PMP   NB:   - Shaded boxes = ≥50% of participants within pattern report it, suggesting prominence of that subtheme for that pattern. - Lighter shading = ≥50% for facilitators; medium shading = ≥50% for barriers; darker shading ≥50% for barriers and facilitators | | | | |
